# Senescence-associated secretory factors induced by cisplatin in melanoma cells promote non-senescent melanoma cell growth through activation of the ERK1/2-RSK1 pathway

**DOI:** 10.1038/s41419-018-0303-9

**Published:** 2018-02-15

**Authors:** Xuerong Sun, Benyan Shi, Huiling Zheng, Ling Min, Jie Yang, Xiaoyi Li, Xiaoxin Liao, Weixing Huang, Mingmeng Zhang, Shun Xu, Zhe Zhu, Hongjing Cui, Xinguang Liu

**Affiliations:** 1 Institute of Aging Research, Dongguan Scientific Research Center, Guangdong Medical University, Dongguan, 523808 China; 2 Guangdong Provincial Key Laboratory of Medical Molecular Diagnostics, Guangdong Medical University, Dongguan, 523808 China; 3 Department of Clinical Laboratory, Affiliated Cancer Hospital & Institute of Guangzhou Medical University, Guangzhou, 51000 China; 4 The Second Clinical School, Guangdong Medical University, Dongguan, 523808 China; 5 School of Laboratory Medicine, Guangdong Medical University, Dongguan, 523808 China; 6 Department of Medicine, Division of Regenerative Medicine, University of California, San Diego, School of Medicine, La Jolla, CA 9500 USA; 7 Institute of Biochemistry & Molecular Biology, Guangdong Medical University, Zhanjiang, 5240238 China

## Abstract

Although targeted therapy and immunotherapy greatly improve the outcome of melanoma, drug resistance and low response rates still maintain the unsubstitutability of traditional chemotherapy. Cisplatin (CDDP) is widely used in different types of tumours with high response rates, but it generally has low efficiency in melanoma. The mechanisms underpinning the phenomena are not sufficiently understood. Here we demonstrated that various melanoma cell lines adopted senescence phenotype after CDDP treatment in contrast to the other types of tumour cells. CDDP treatment induced melanoma A375 cells into senescence through the sequential activation of the DNA damage response and the P53/P21 pathway. All the senescent melanoma cells induced by CDDP alone or the combination of CDDP and dacarbazine developed robust senescence-associated secretory phenotype (SASP), that is, the secretion of multiple cytokines. IL-1α was an early component and an upstream regulator of SASP. Similarly, CDDP either alone or combined with dacarbazine could induce melanoma cell senescence and SASP in either A375 or B16F10 melanoma xenograft mice. The supernatant of senescent A375 cells promoted the growth of normal non-senescent A375 cells and enhanced their expression and secretion of IL-8 through the activation of the ERK1/2-RSK1 pathway. The transplantation of non-senescent and senescent A375 cells together into nude mice showed accelerated tumour growth compared with transplanting non-senescent cells alone; no tumours developed when transplanting senescent cells alone. Following CDDP administration in A375-bearing mice, the intratumour injection of neutralisation antibodies targeting the SASP factors IL-1α or IL-8 evidently delayed tumour growth. The results suggest that the CDDP-induced senescent melanoma cells promote non-senescent cells proliferation through the activation of ERK1/2-RSK1 pathway by the SASP factors. Cell senescence and concomitant SASP may be the particular mechanisms for melanoma to resist chemotherapeutics.

## Introduction

Melanoma consistently shows increased incidence almost all over the world^1^. The established risk factors for melanoma include family history, multiple moles, fair skin, ultraviolet radiation and immunosuppression^2^. Some of the risk factors, especially ultraviolet radiation, can lead to somatic base mutation. BRAF^V600E^ is the most common mutation site, occurring in about 50% of patients and resulting in the hyperactivation of the MAPK pathway.

Drug therapy is essential for metastatic melanoma. The traditional chemotherapeutic drugs, such as cisplatin, dacarbazine and paclitaxel (PTX), are generally low in efficiency. In recent years, the targeted inhibitors of BRAF (vemurafenib) or MEK (binimetinib) have shown improved survival and response rates in metastatic melanoma^3–5^. Alternatively, immunotherapies have made great breakthroughs. Immune checkpoint inhibitors, such as PD-1 antibody and CTLA-4 antibody, produce striking durable responses and curative outcomes^2,6^. Nevertheless, both targeted therapies and immunotherapies have obvious limitations, such as drug resistance and improved but still low response rates^7,8^. Immunotherapies can even hasten the spread of cancer in some patients^9^. Therefore, traditional chemotherapies are still indispensable in melanoma therapy ^10^.

Cisplatin (CDDP, cis-Diaminodichloroplatinum) is one of the most widely used chemotherapeutic agents^11,12^. In the latest guideline recommended by the National Comprehensive Cancer Network, CDDP is consistently regarded as the first-line agent against lung cancer and cervical cancer, among others. However, melanoma is inherently resistant to CDDP, and the mechanisms are not fully understood. In this study, we investigated the effect of CDDP on several types of tumour cells and revealed that melanoma is particularly inclined to enter into senescence. The cell senescence and concomitant senescence-associated secretory phenotype (SASP) may be the usual mechanisms underlying the resistance of melanoma to chemotherapy.

## Results

### CDDP-induced robust cell senescence in melanoma A375 cells through the P53/P21 pathway

**Fig. 1:**
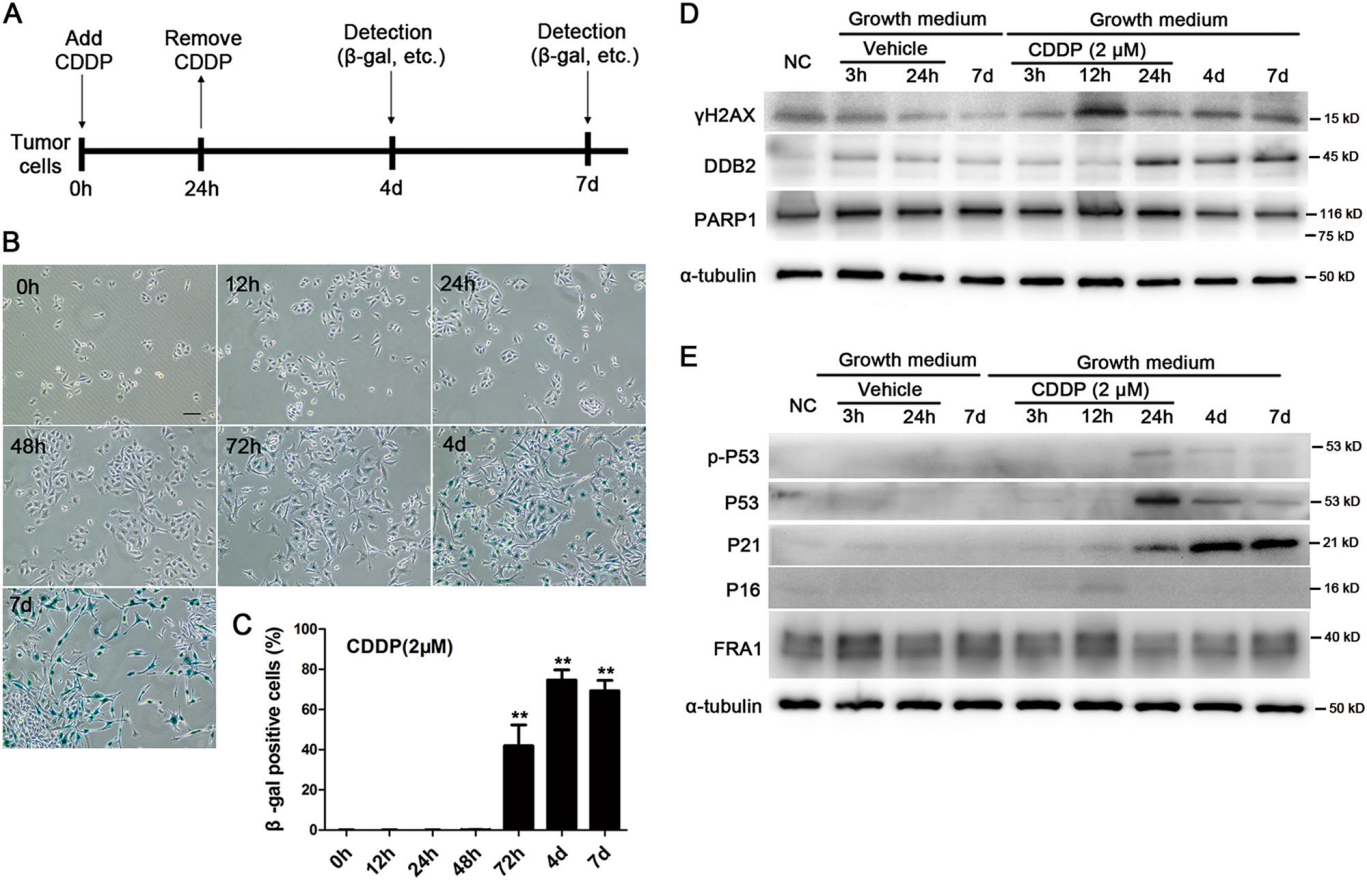
CDDP-induced A375 cell senescence through DNA damage response and the P53/P21 pathway. **a** The general protocol used to induce cell senescence, that is, tumour cells were treated with CDDP for 24 h, and then various detections were performed at different time points. **b**, **c** After treatment with 2 μM CDDP, the activity of senescence-associated β-gal was evaluated from 12 h to 7 days (**d**), and the ratios of β-gal-positive (blue-stained) cells were calculated (*n* = 3). **d**, **e** Expression of proteins related to DNA damage (γH2AX and DDB2), apoptosis (PARP1), senescence (P53, P21 and P16) or drug resistance (FRA1) in A375 cells after CDDP treatment were detected by western blot. The bar in **b** is 100 μM. **P < 0.01 vs. 0 h

To observe the effect on melanoma, CDDP was added to the growth medium of A375 cells (defined as 0 h) at various final concentrations. 24 h later, CDDP was removed and detections were performed at different time points (Fig. 1a). After the CDDP treatment, an enlargement of the cellular morphology was observed, thus implying cell senescence. Thus, the activity of senescence-associated β-galactosidase (β-gal), a canonical marker of cell senescence, was evaluated. 4 days after the CDDP treatment, the β-gal-positive (blue-stained) cells were observed when CDDP was greater than 2 μM (Supplementary Fig. 1A). On the seventh day, the blue colour deepened, which implied a stable cell cycle arrest in the stained cells (Supplementary Fig. 1B). Note that in 2 μM CDDP, a few cells escaped from senescence and formed proliferative clones on the seventh day. In 4 or 10 μM CDDP, most of the survival cells became senescent and few clones were observed. A weak cleaved band of PARP1 protein, an apoptosis marker, could only be detected in 10 μM CDDP (data not shown). In the following experiments, 2 or 4 μM CDDP was used unless otherwise indicated.

The time-dependent development of cell senescence was evaluated. After the CDDP treatment, the enlargement of cell morphology was initiated as early as 12 h and kept on increasing up to 4 days. The blue-stained senescent cells appeared as early as 72 h and became overwhelming after 4 days (Fig. 1b, c).

To explore the mechanisms involved into cell senescence, the expression levels of DNA damage response (DDR)-related proteins (γ-H2AX and DDB2), PAPR1 and senescence-related proteins (P53/P21 and P16/pRb), among others, were continuously monitored after the CDDP treatment. The results showed that the increase of γ-H2AX occurred first, followed by DDB2 (a key protein involved in DDR), p-P53 and P53, and then by P21. The upregulation of P21 was maintained up to 7 days (Fig. 1d, e). The FRA1 protein participating in vemurafenib resistance in melanoma^13^ showed no evident change. Quantitative reverse transcription PCR (qRT–PCR) showed that, CDDP significantly increased the mRNA expression of P21 but not that of P53 or P16 (Supplementary Fig. 2A). At 7 days after the CDDP treatment, immunofluorescence revealed the obvious accumulation of P21 in the cell nuclei, with the strongest fluorescence located in the huge, malformed nuclei, a typical characteristic of cell senescence (Supplementary Fig. 2B). The results suggested that the activation of the P53/P21 pathway, mediated by the DNA damage response, could underlay the senescence of A375 cells as reported^14,15^.

### CDDP did not induce evident cell senescence in other types of tumour cells

**Fig. 2:**
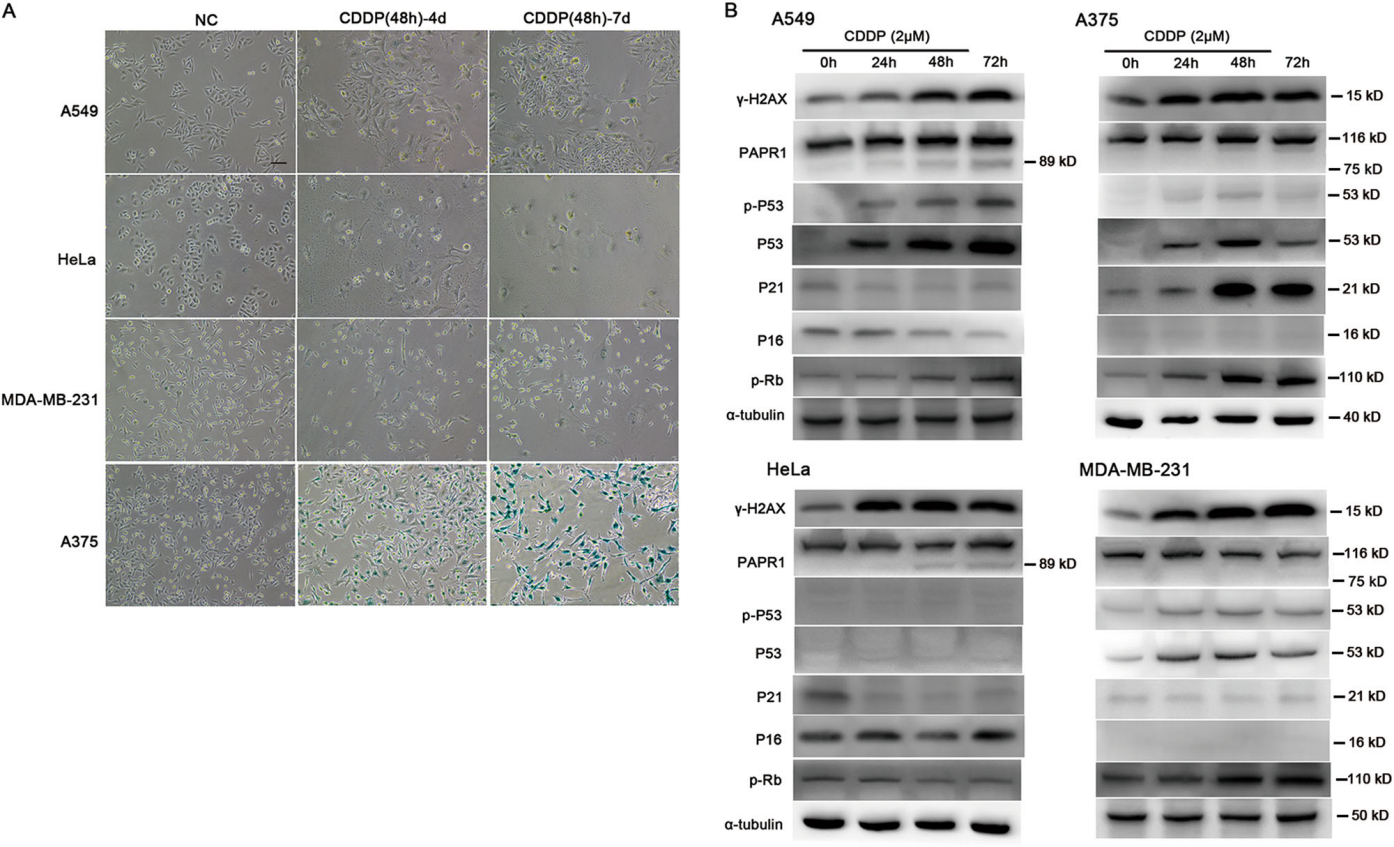
Differential induction of senescence and senescence-related proteins in different tumour cells after the CDDP treatment. The tumour cells used here included lung cancer A549 cells, cervical carcinoma HeLa cells, breast carcinoma MDA-MB-231 cells and melanoma A375 cells. **a** CDDP (2 μM) was added to the culture medium and removed after 48 h. β-gal staining was performed on the fourth or seventh day. **b** Dynamic expressions of the proteins related to DNA damage response, apoptosis and senescence were detected in these tumour cells after the CDDP treatment. The bar is 100 μm in **a**. Note that CDDP-induced robust cell senescence and activated the P53/P21 pathway exclusively in A375 cells. A cleaved band of PARP1 (89 kD) could be observed in A549 and Hela cells. Similar results were obtained in three independent experiments

Although the enlargement of cell morphology could be easily observed in lung cancer A549, cervical carcinoma HeLa and breast carcinoma MDA-MB-231 cells, few blue-stained cells could be discerned after the CDDP treatment at various concentration (Supplementary Fig. 3). To further confirm the results, the duration of the CDDP treatment was prolonged to 48 h. The results consistently demonstrated robust β-gal staining in A375 cells but not in other types of tumour cells (Fig. 2a).

To reveal the molecular basis underpinning the differential responses to CDDP, DDR- and senescence-related proteins were detected in the four types of tumour cells. The results showed that CDDP increased the γ-H2AX expression in all tumour cells, thus implying similar DNA damage (Fig. 2b). Although the upregulation of P53 and p-P53 proteins was evident in A375, A549 and MDA-MB-231 cells, a robust increase in P21 occurred exclusively in A375 cells. No upregulation of P16 was observed, and the upregulation of p-Rb occurred in A375, A549 and MDA-MB-231 cells. The apoptotic cleavage fragments of PARP1 were only observed in A549 and HeLa cells, consistent with the preferable effect of CDDP on these tumours. The results further supported a key role of P53/P21 pathway activation in A375 cell senescence.

### CDDP-induced robust SASP in melanoma A375 cells

**Fig. 3:**
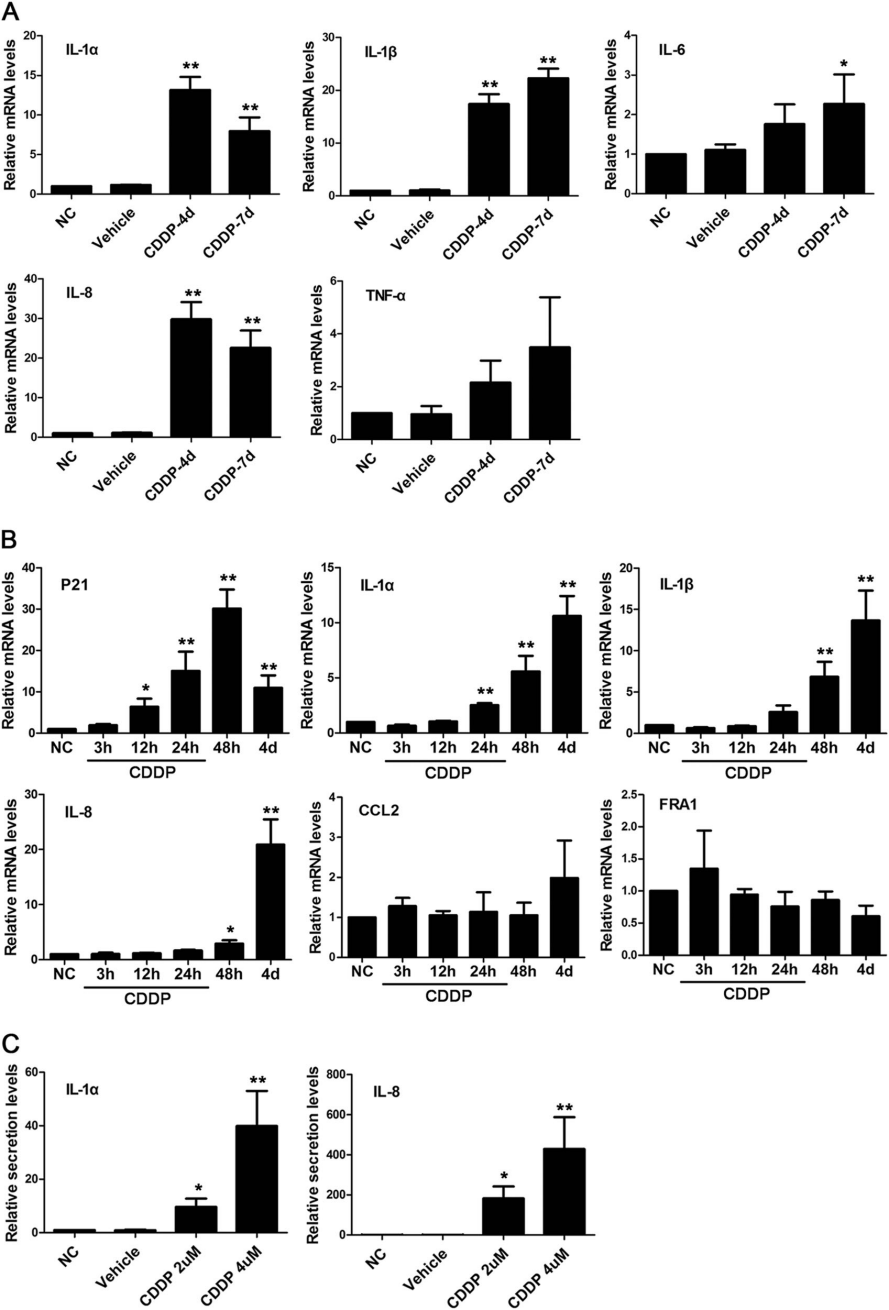
CDDP-induced robust SASP in A375 cells. **a** CDDP (2 μM) treatment enhanced the mRNA levels of SASP genes after 4 or 7 days. Vehicle-treated cells were collected and detected after 7 days. Normal A375 cells served as the negative control (NC). **b** Time-dependent expressions of P21, the SASP genes and vemurafenib resistance-related gene (FRA1) after the CDDP treatment (2 μM). **c** DMEM containing 0.5% FBS was added into the dishes at 4 days after the CDDP treatment, and the conditioned medium (CM) was collected 24 h later. ELISA detection showed that the secretion of IL-1α and IL-8 evidently increased after the CDDP treatment in comparison with the vehicle or normal culture. Data were from three independent experiments. *P < 0.05, **P < 0.01 vs. NC (*n* = 3)

To determine whether the senescent A375 cells developed SASP, we identified the mRNA levels of several SASP genes that exhibited frequent upregulations in other senescent cells^16,17^. The results demonstrated that at 4 or 7 days after the CDDP treatment, the mRNA levels of IL-1α, IL-1β, IL-6, IL-8 and TNF-α consistently increased (Fig. 3a). To clarify the chronological relationship between senescence and SASP, we determined the dynamic expression of P21 and SASP genes by qRT–PCR. The results showed that P21 increased first, followed by IL-1α and IL-1β. The robust increase in IL-8 occurred last on the fourth day (Fig. 3b).

Although CCL2 was reported as an important component of SASP in senescent melanoma cells induced by microphthalmia-associated transcription factor silencing^18^, its upregulation was limited in A375 cells in this study (Fig. 3b). Likewise, the robust downregulation of FRA1 has been regarded as a key driver of vemurafenib-induced secretome in A375 cells^13^, but only a weak decrease was observed at the mRNA level in this study (Fig. 3b). These results strengthened the view that SASP is highly context dependent^17^.

Immunofluorescence showed that senescent A375 cells exhibited an enhanced expression of IL-8 in the cytoplasm (Supplementary Fig. 2B). Then, the supernatant of A375 cells was harvested on the fifth day after the CDDP treatment (2 or 4 μM) and used for ELISA detection. The results showed that CDDP obviously enhanced the secretion of IL-8 and IL-1α, with a stronger effect in 4 μM than 2 μM CDDP (Fig. 3c). All the results suggested that CDDP enhanced the expression of SASP genes at both mRNA and protein levels and that the SASP factors could be secreted into extracellular fluid.

### Cell senescence and SASP were common responses of melanoma cells to CDDP-based treatment

Cell senescence and SASP were further investigated in human A375 (BRAF^V600E^ mutation) and mice B16F10 (BRAF wild) melanoma cells using two more chemotherapeutic drugs, namely, PTX and dacarbazine. The β-gal staining and qRT–PCR showed that PTX could induce evident cell senescence and SASP in both cell lines (Supplementary Fig. 4). However, dacarbazine could not enhance the activity of β-gal in these cells, although an aberrant increase in some SASP genes was observed (Supplementary Fig. 5).

Induction of senescence and SASP by CDDP were further explored in 5 other melanoma cell lines, including mice B16F10 and human A875, OCM-1A, SK-MEL-2 and M14 cells. CDDP treatment promoted β-gal staining in these cells over a wide range of concentrations (data not shown). Besides β-gal staining, the treated cells displayed enhanced expression of P21 and SASP genes, as well as increased secretion of IL-8 (Supplementary Fig. 4, Supplementary Fig. 6).

Since CDDP is generally combined with other drugs in melanoma treatment, we further confirmed that the combination of CDDP and dacarbazine could also induce senescence and SASP in four randomly selected melanoma cell lines, including A375, A875, M14 and SK-MEL-2 (Supplementary Fig. 7).

### IL-1α was an upstream regulator of SASP in senescent A375 cells

**Fig. 4:**
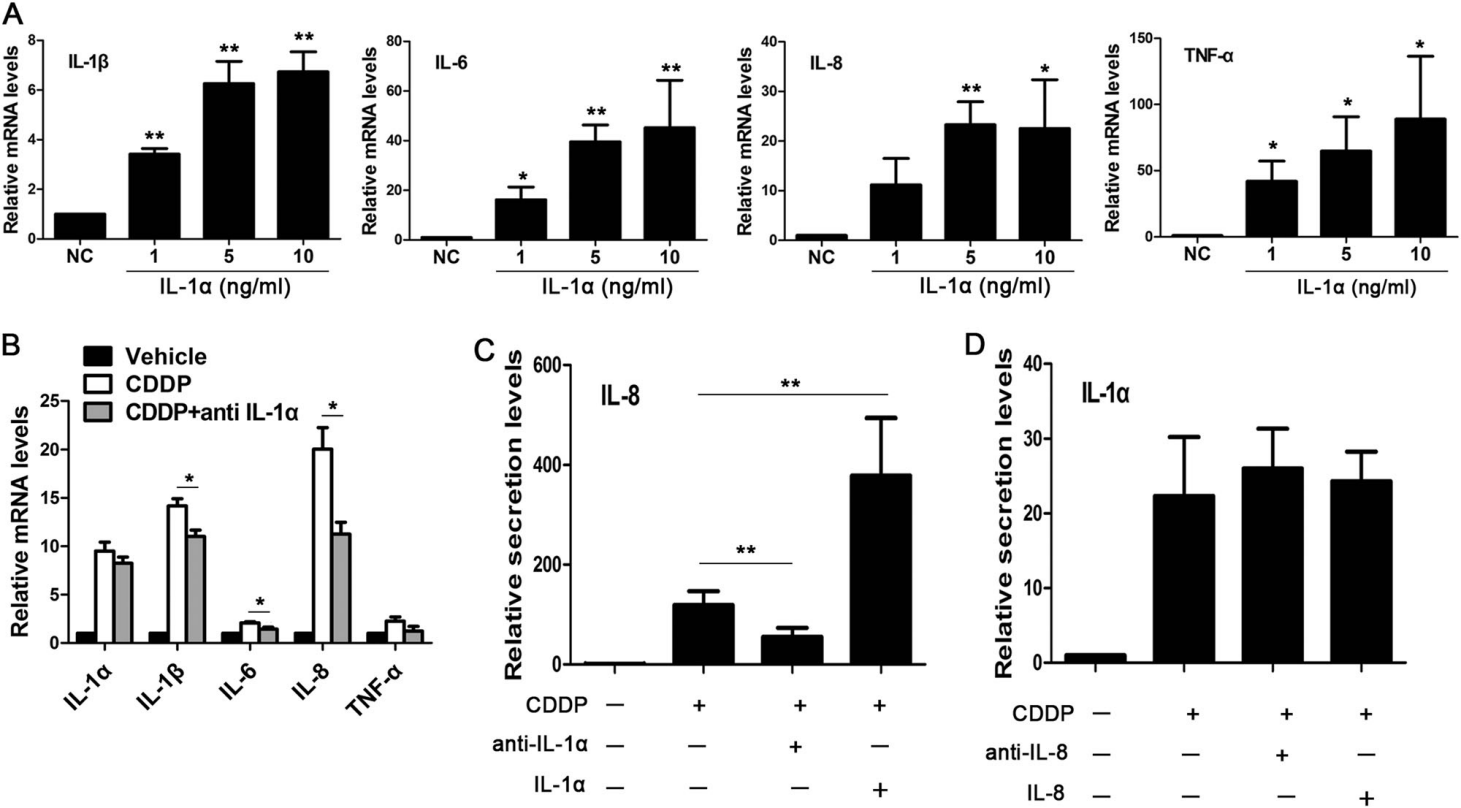
IL-1α was an upstream regulator of SASP in senescent A375 cells. **a** After treatment of normal non-senescent A375 cells with IL-1α for 24 h, the expressions of IL-1β, IL-6, IL-8 and TNF-α increased in a concentration-dependent manner. **b**–**d** On the fourth day of CDDP treatment (2 μM), the antibodies (anti-IL-1α and anti-IL-8) or cytokines of IL-1α and IL-8 were added to the growth medium, respectively. After 24 h of incubation, the mRNA level of *SASP* genes was quantified by qRT–PCR (**b**), and the secretion levels of IL-8 (**c**) and IL-1α (**d**) were detected by ELISA. Data were from three independent experiments. *P *< *0.05, **P < 0.01 vs. NC or between the indicated two groups (*n* = 3)

IL-1α is not only an early component but also an upstream regulator of SASP in human fibroblasts^19,20^. To explore the possible regulatory role of IL-1α in A375 cells, we first explored its effect on normal A375 cells. qRT–PCR showed that IL-1α increased the expression of IL-1β, IL-6, IL-8 and TNF-α in a concentration-dependent manner (Fig. 4a).

To investigate whether IL-1α was actually involved in the regulation of other SASP factors in senescent A375 cells, the neutralisation antibody of IL-1α (anti-IL-1α) was added to the growth medium on the fourth day after the CDDP treatment. 24 h later, qRT–PCR showed that anti-IL-1α (2.5 μg/ml) uniformly decreased the mRNA levels of IL-1β, IL-6, IL-8, TNF-α and IL-1α (Fig. 4b). The secretion levels of IL-8 also significantly decreased (Fig. 4c). The addition of the cytokine IL-1α to the senescent cell culture further increased the secretion of IL-8. On the contrary, when the neutralisation antibody (anti-IL-8) or the cytokine of IL-8 was added to the growth medium, both exerted no effect on IL-1α secretion (Fig. 4d).

### CDDP-based treatment induced senescence and SASP in melanoma xenograft mice

**Fig. 5:**
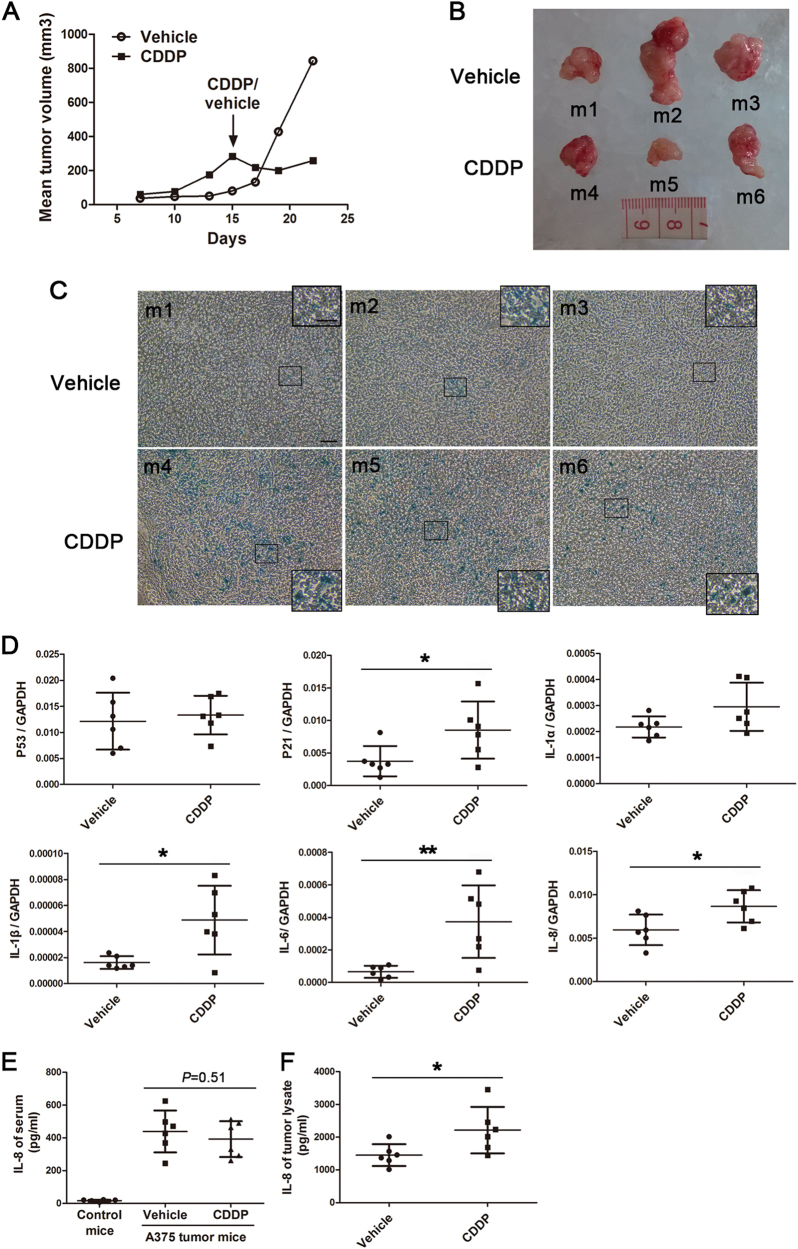
CDDP-induced senescence and SASP in A375 tumour xenograft mice. Upon tumour formation in nude mice at 15 days after the subcutaneous transplantation of A375 cells, the CDDP or the vehicle was delivered intraperitoneally for once. Tumour tissues and serum were collected after another 7 days. **a** CDDP treatment regressed tumour growth transiently while the vehicle was incompetent. The mean tumour volume was derived from six mice in each group (*n* = 6). **b** Representative pictures of A375 tumour tissues after the CDDP or vehicle treatment. m1–m6 were the mice number. **c** CDDP treatment enhanced β-gal staining in tumour frozen sections. The bar is 100 μM in the primal graph and 50 μM in the inset. **d** Tumour lysates from CDDP-treated mice showed higher mRNA levels of P21 and SASP genes than those from vehicle-treated mice. **e** CDDP treatment did not affect serum IL-8 concentration in tumour-bearing mice compared with the vehicle. Normal mice without cell grafting served as the control (*n* = 6). **f** CDDP treatment elevated the IL-8 expression in tumour lysates evaluated by ELISA. * *P* < 0.05; ** *P* < 0.01

To investigate whether CDDP could induce cell senescence and SASP in vivo, we made tumour xenografts in nude mice through the subcutaneous injection of A375 cells. After the appearance of an obvious tumour mass, the CDDP or vehicle solution was intraperitoneally injected. A week later, the tumour tissues were harvested and detected. The results showed that a single-dose injection of CDDP could reduce tumour volume transiently (Fig. 5a, b). The β-gal staining of tumour frozen sections showed that CDDP evidently increased the ratios of blue-stained cells compared with the vehicle (Fig. 5c). qRT–PCR demonstrated that the tumour tissue in the CDDP group showed an increased expression of P21 and SASP genes, such as IL-1β, IL-6 and IL-8 (Fig. 5d). ELISA further confirmed the enhanced concentration of IL-8 in the CDDP-treated tumour lysates compared with the vehicle-treated ones (Fig. 5f). However, the serum concentration of IL-8 showed no obvious difference between CDDP- and vehicle- treated mice (Fig. 5e). The results suggested that the CDDP-induced IL-8 exerted the physiological effect mainly in tumour microenvironment.

**Fig. 6:**
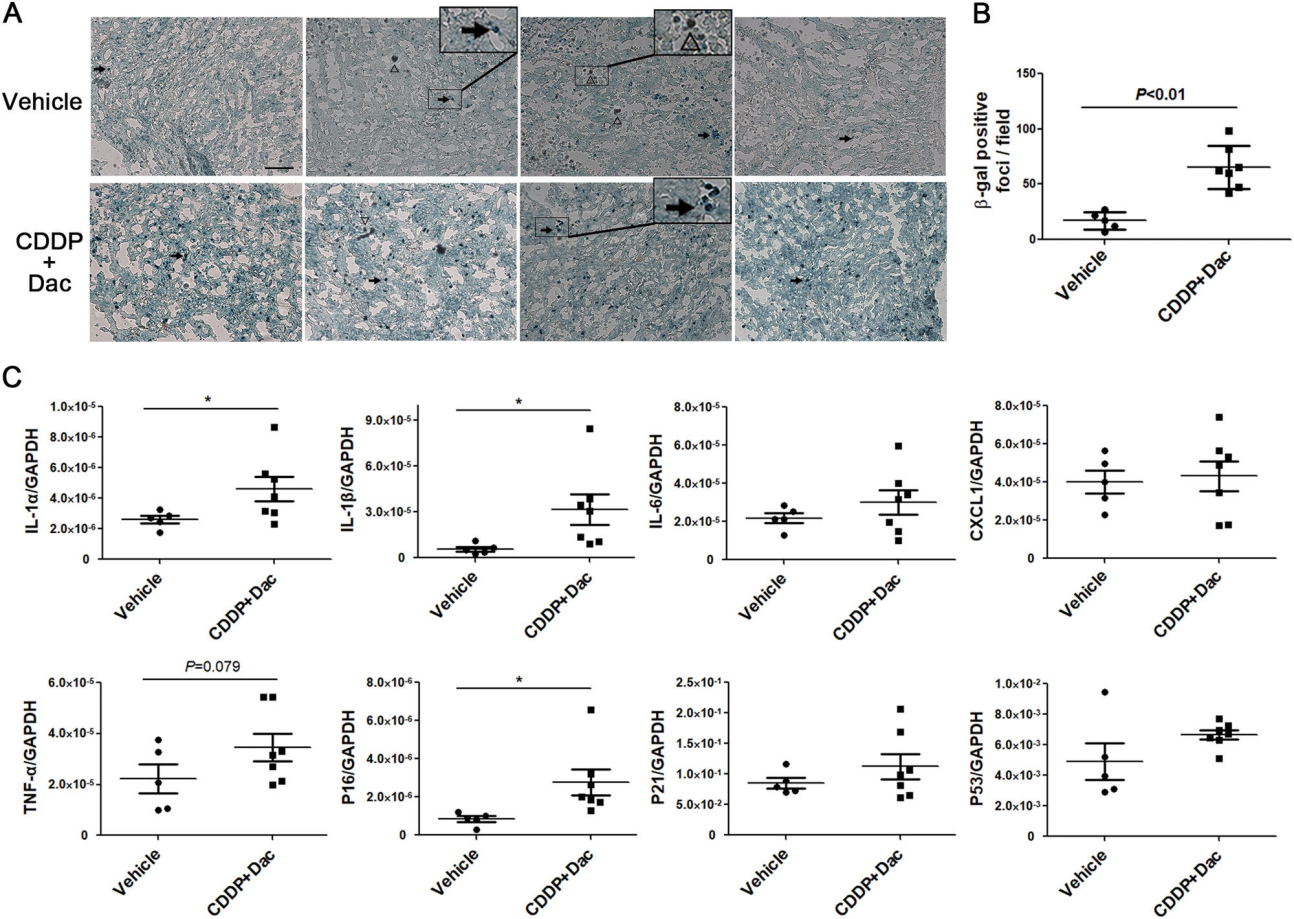
CDDP together with dacarbazine induced cell senescence and SASP in B16F10 tumour xenograft. After the formation of tumour in C57BL/6 mice, both CDDP and dacarbazine (Dac) were intraperitoneally injected twice. **a**, **b** β-gal staining of frozen tumour slices 5 days after CDDP and dacarbazine administration. Each picture represents an individual mouse. The representative pictures of four mice were shown for each group. The blue-stained foci, indicated by arrow, were significantly more abundant in 'CDDP + Dac' mice (*n* = 7) than those of vehicle mice (*n* = 5). The triangles indicate the melanin granules. The bar is 200 μM. **c** The relative mRNA levels of SASP and senescence-related genes in tumour lysates of the two groups. *P < 0.05

The synthetical effect of CDDP and dacarbazine on melanoma was further investigated in C57BL/6 mice with intact immune system. When tumour masses were evident about 1 week after B16F10 cells injection, both dacarbazine and CDDP were intraperitoneally administrated twice (Supplementary Fig. 8). After another 5 days, β-gal staining of frozen tumour sections indicated that, the combined treatment induced numerous blue-stained foci (Fig. 6a, b). qRT–PCR showed that the combined treatment generally increased the expression of SASP and senescence-related genes in tumour tissues (Fig. 6c).

### The SASP factors promoted the growth of A375 cells in vitro by the activation of ERK1/2-RSK1 pathway

**Fig. 7:**
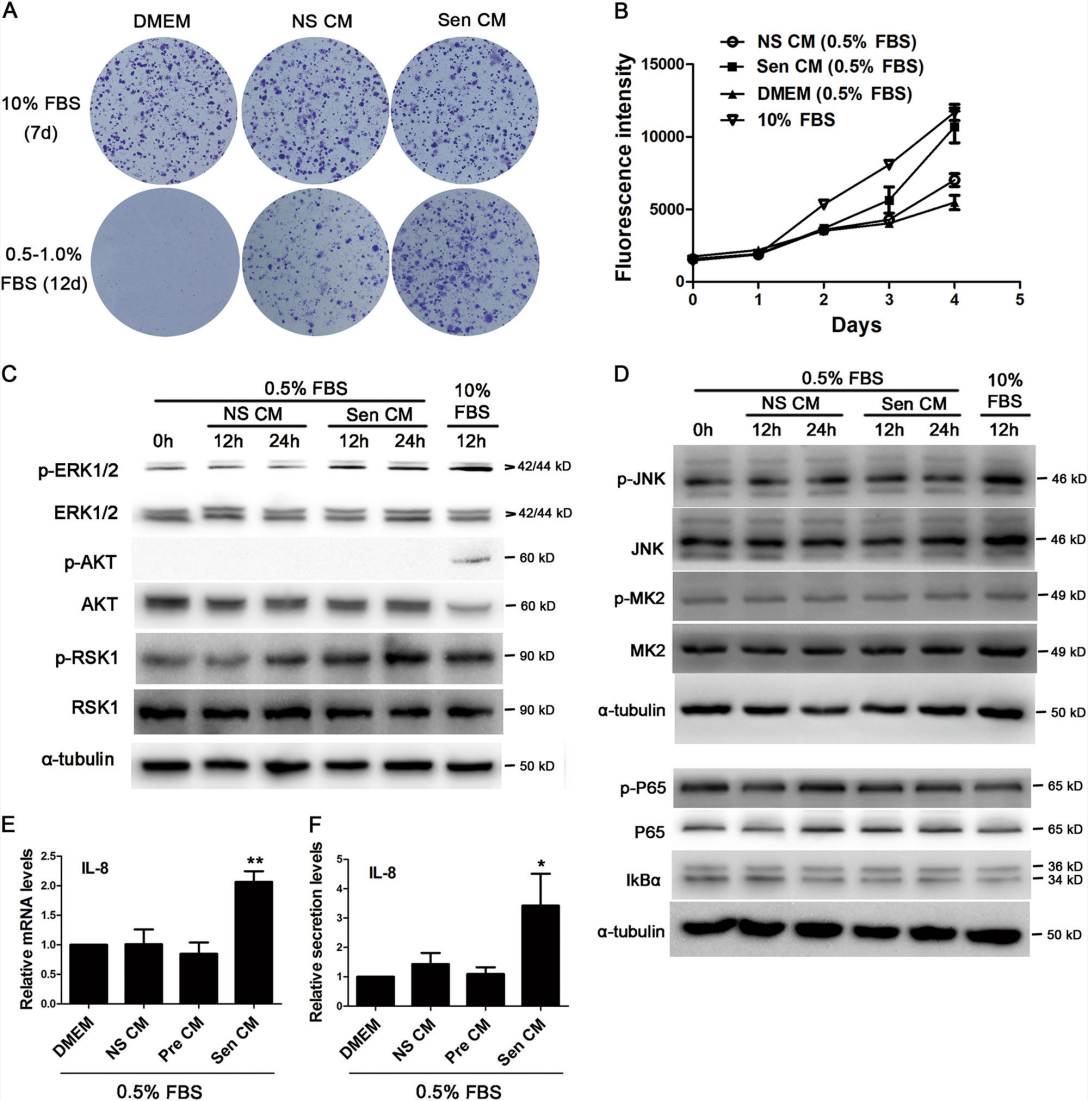
The conditioned medium (CM) of senescent A375 cells promoted the non- senescent cell growth probably through the activation of the ERK1/2-RSK1 pathway. The non-senescent (NS), senescent (Sen) and pre-senescent (Pre) CMs were harvested after the vehicle or CDDP (4 μM) treatment according to Methods. DMEM or the CMs were premixed with FBS-containing DMEM (1:1) to arrive at a final serum concentration of 0.5, 1 or 10%. Except for CFA, normal A375 cells were serum starved for 48 h before being treated with CM. **a** Sen CM enhanced the clone formation ability of A375 cells in 0.5–1% FBS conditions compared with NS CM and DMEM. Similar results were obtained from three independent experiments. **b** Sen CM promoted A375 cell proliferation in 0.5% FBS conditions, evaluated by the alamarBlue assay. The values were mean ± SEM of five parallel wells. Similar results were obtained from three independent experiments. **c**, **d** Sen CM promoted the phosphorylation of ERK1/2 and RSK1 but exerted no effect on p-AKT, total AKT, p-JNK, total JNK, p-MK2, total MK2, p-P65, total P65 and IκBα. **e**, **f** DMEM or various CMs were incubated with A375 cells for 24 h, and the mRNA levels of IL-8 were evaluated by qRT–PCR (*n* = 3) (**e**). Alternatively, the medium was completely replaced by 0.5% FBS medium. After another 24 h, the IL-8 secretion level in the supernatant was detected by ELISA (**f**). *P < 0.05, **P < 0.01 *vs*. DMEM (*n* = 3)

To explore the physiological role of SASP factors in tumour growth, the conditioned media (CM) from senescent (Sen) and normal non-senescent (NS) A375 cells were harvested and added to the culture medium of normal A375 cells. In low fetal bovine serum (FBS) (0.5%) conditions, both CMs enhanced the colony formation ability of A375 cells compared with Dulbecco’s modified Eagle’s medium (DMEM), and Sen CM showed a stronger effect than NS CM (Fig. 7a). Similarly, although both Sen and NS CMs could promote the proliferation of A375 cells, a stronger effect was observed in Sen CM (Fig. 7b).

To reveal the signal pathway activated by Sen CM, we identified several pathways regulated by IL-8^21^ or IL-1α. When added into serum-starved A375 cells, Sen CM activated the ERK1/2-RSK1 pathway by enhancing the phosphorylation of ERK1/2 and RSK1, while NS CM seemed incompetent (Fig. 7c). Both CMs showed no obvious effect on the AKT, JNK, p38-MK2 and NF-κB pathways (Fig. 7c, d). Similarly, Sen CM failed to induce the obvious nuclear translocation of P65 protein after a 0.5 or 2 h treatment, as determined by immunofluorescence (data no shown).

Then mRNA array was employed to investigate the special genes regulated by Sen CM. When DMEM (NC), NS CM or Sen CM was added into serum-starved A375 cells, very limited differential genes were revealed with a fold change greater than two times (Supplementary Fig. 9). Relative to NC, both Sen and NS CMs induced the same upregulation of SLC30A1 and downregulation of TAC1. In comparison with NS CM, Sen CM enhanced the expression of IL-8 and suppressed the expressions of MEF2C, SOCS2 and SPON2 (Supplementary Fig. 9). qRT–PCR and ELISA further confirmed that Sen CM promoted A375 cells to express and secrete IL-8 (Fig. 7e, f). When differential genes with a fold change greater than 1.5 times were analysed, 19 genes were upregulated and 12 genes were downregulated by Sen CM in comparison with NS CM. More than half of these genes were related to the ERK1/2 pathway and showed a generally consistent change with the activation of the pathway (Supplementary Table 1).

As not all cells became senescent after the CDDP treatment, the CM of pre-senescent cells (Pre CM) were collected at 2 days after the CDDP treatment to verify that the cytokines promoting the IL-8 expression came from real senescent cells. Further observation showed that Pre CM did not affect the expression or the secretion of IL-8 (Fig. 7e, f). The results suggested that the effective cytokines came from fully senescent cells.

### Senescent A375 cells promoted the proliferation of non-senescent cells in vivo through the SASP factors

**Fig. 8:**
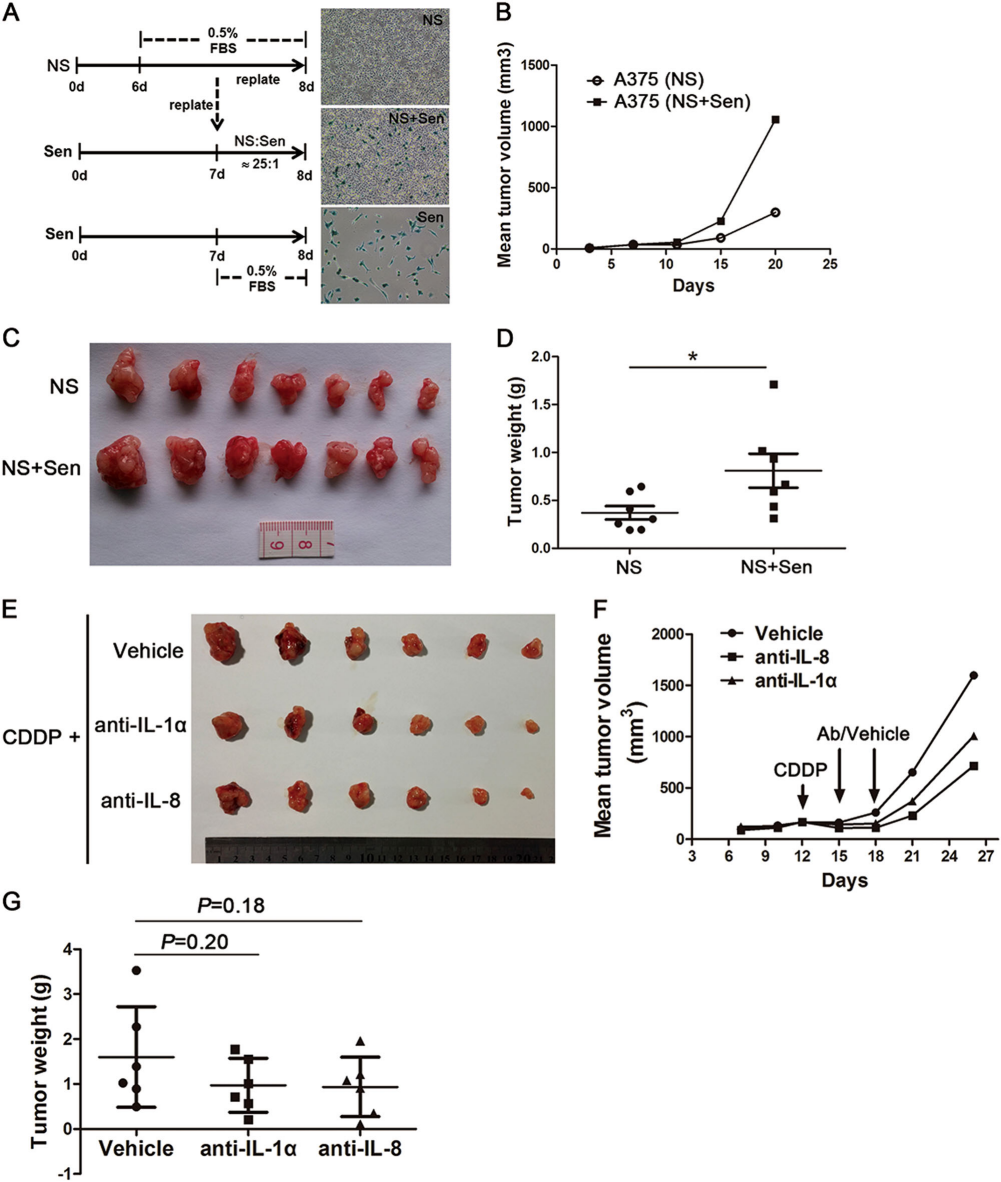
Senescent A375 cells promoted the non-senescent cell proliferation in vivo probably through the secretion of IL-1α and IL-8. **a**–**d** Exploring the effect of senescent cells on non-senescent cells in vivo. **a** After 24 h of serum starvation, NS cells were divided into two parallel parts and seeded into either empty or senescent cell-containing dishes. After another 24 h of culture in low serum conditions, the same amount (4 × 10^6^) of ‘NS’ or ‘NS + Sen’ cells was transplanted into mice (*n* = 7 for each group). **b** Time-dependent development of the mean tumour volume in A375 xenograft mice. **c**, **d** Tumour morphology and weights in the two groups of mice. To facilitate the comparison, the tumour tissues in each group were arranged according to size. **e**–**g** Determination of whether the neutralising antibody (Ab) delivery following CDDP could delay tumour growth. CDDP was intraperitoneally administrated, and the neutralising antibodies (anti-IL-1α or anti-IL-8) or vehicle was intratumour injected twice, respectively (*n* = 6 for each group). **e** Tumour tissues from vehicle-treated or antibody-treated mice. **f** Drug delivery (indicated by arrow) and continuous monitoring of the mean tumour volume. **g** Final tumour weights in the three groups of mice. *P < 0.05

Nude mice were used to investigate whether senescent A375 cells could enhance the proliferation of NS cells in vivo. To strengthen the effect of Sen cells, NS A375 cells were serum starved for 24 h and then seeded into dishes containing Sen cells resulting from the CDDP (6 μM) treatment. After another 24 h co-culture in a low serum medium, the mixture containing both NS and Sen cells was transplanted (Fig. 8a). The injected cell amount (NS + Sen) was 4 × 10^6^ cells per mouse. The same quantity of NS A375 cells (4 × 10^6^) was transplanted as the control. The results showed that the tumours in NS + Sen mice grew faster and produced significantly larger masses than those in NS mice (Fig. 8b–d). Either the 1.6 × 10^5^ (which is equal to the Sen cell number in ‘NS + Sen’) or the 1 × 10^6^ Sen cells were transplanted into nude mice (*n* = 3 for each group) to explore their tumourigenicity. In all the six mice, no tumour tissue developed in 3 weeks (data not shown). On the contrary, when 1 × 10^6^ NS cells were transplanted, all the mice (*n* = 3) produced evident tumour mass (data not shown). These results suggested that the Sen cells in NS + Sen could hardly form tumour by themselves, but they could promote the proliferation of NS cells.

IL-8 and IL-1α generally exert a positive effect on cell proliferation^22,23^, as verified in A375 cells under low serum conditions (Supplementary Fig. 10). As senescent A375 cells expressed high levels of IL-8 and IL-1α, we further explored if the neutralising antibodies targeting IL-8 or IL-1α could improve the therapeutic effect of CDDP. At three and 6 days following the CDDP injection in tumour-bearing mice, the IL-8 or IL-1α antibody was intratumour injected twice, independently (Fig. 8f). The results showed that both antibodies delayed tumour growth. The mean tumour weight decreased from 1.6 g in vehicle-treated mice to 0.97 and 0.94 g in anti-IL-1α-treated and anti-IL-8-treated mice, respectively (Fig. 8e–g). Although the differences were not significant because of the small sample size, the decrease was evident. The results suggested that the inhibition of key SASP factors could improve the therapy effect of CDDP on melanoma.

## Discussion

Cell senescence was first described by Hayflick, and it refers to an irreversible cell cycle arrest after a maximum of 50 cell divisions in vitro^24^. This phenomenon is now known as ‘replicative senescence’. Various types of stress can also lead to senescence, which is referred to as ‘stress-induced premature senescence’. Generally, senescent cells exhibit enlarged morphology, enhanced activity of β-galactosidase and activation of the P53/P21 or P16/pRB pathway. Although growth arrested, senescent cells usually show strong secretion activity, which is known as SASP. The SASP factors include a wide range of growth factors, proteases and chemokines, which are generally regulated at the mRNA levels^16,17^.

DNA damage signalling plays an essential role in cell senescence. CDDP usually causes Pt-DNA adducts and indirectly leads to DNA double-strand breaks, which are repaired by nucleotide excision repair^25^, non-homologous end joining (NHEJ) or homologous recombination (HR). PTX is a microtubule-targeted drug that can also generate free radicals^26^ and induce DNA breaks, thus triggering DNA repair pathways, such as base excision repair (BER), NHEJ and HR^27^. Dacarbazine can cause DNA damage by producing O6-methylguanine and N7-methylguanine in genomic DNA, which usually trigger MGMT (O6-MethylguanineDNA methyltransferase) repair, mismatch repair or BER^28,29^. In the current study, CDDP and PTX induced evident senescence and SASP, whereas dacarbazine exhibited a weak effect. This result implies that the specific patterns of DNA damage or DNA repair have a great influence on senescence. Furthermore, dacarbazine needs to be activated in vivo to exert the cytotoxic effect^30^. The in vitro light activation used in this study could not have completely mimicked the metabolite of dacarbazine.

The study demonstrated that CDDP-based treatment could induce cell senescence and SASP in melanoma cells in vitro and in vivo. The reasons why other tumour cells, such as A549, HeLa and MDA-MB-231 cells, could not be easily induced into senescence could be related to their genomic backgrounds. The mutant P53 (R280K) in MDA-MB-231 cells^31^ may contribute to the failure in activating P21. HeLa cells have a compromised P53 because of the accelerated degradation by the virally encoded E6 protein^32^. It has been reported that a higher concentration of CDDP can enhance P21 and P53 expression in A549^33^ and HeLa cells^32^, respectively. Nevertheless, our observation in A549 cells showed that few cells were β-gal positive even if P21 expression was elevated by a higher concentration of CDDP (data no shown).

Both senescence and SASP are multistep and temporally regulated programmes^17^. The current study showed that, after CDDP treatment, the upregulation of P21 mRNA in A375 cells preceded that of IL-1α and IL-1β, which was followed by the enhanced expression or activity of IL-8 and β-gal. The results were consistent with the existence of an early self-amplifying SASP and a late mature SASP^17^.

Our in vitro study demonstrates that senescent CM can promote the growth of non-senescent A375 cells. In in vivo experiments, senescent cells have been reported to collapse quickly after being implanted into SCID mice because of a non-selective adaptation and the selective clearance by macrophage^34^. To minimise the disadvantage caused by the clearance of senescence cells in vivo, NS A375 cells were pre-activated by co-culturing with Sen cells before being transplanted. The results demonstrated that Sen A375 cells could promote the growth of NS cells in nude mice. However, the results did not distinguish whether Sen cells exerted the effect in vitro, in vivo or both. Therefore, we further investigated the problem by neutralising the SASP factors in vivo. When neutralising antibodies were intratumour delivered after SASP induction, they clearly delayed tumour growth. The results imply an active role of SASP factors in tumour microenvironment and further suggest that IL-1α and IL-8 are the key SASP factors promoting the growth of NS cells. The cytokine CCL2-induced^18^ and FRA1-induced secretosome^13^ may not be the key factors in this study.

Among the detected signal pathways, senescent CM specifically enhances the phosphorylation of ERK1/2 and the downstream RSK1. Actually, many of the SASP factors, including IL-1α^35^, IL-1β^36^ and IL-8^22^, can activate the ERK1/2 pathway. Further detection confirmed that Sen CM enhanced the expression and secretion of IL-8, which is a downstream effector of the ERK1/2 pathway. Therefore, the IL-8 releasing from senescent cells probably induced a reproductive positive feedback in adjacent non-senescent cells by activating the ERK1/2-RSK1-IL-8 pathway^37^. Although activated ERK1/2 may promote cell death or cell survival depending on the context, the phosphorylated RSK1 acts as a pro-survival signal in cell growth^38^. In summary, the current study suggests that the activation of the ERK1/2-RSK1 pathway, followed by IL-8 accumulation, is the underpinning mechanism by which the Sen cells promotes the proliferation of NS cells. As IL-1α is the upstream regulator of SASP, its effect may be partially mediated by IL-8.

Aside from CDDP and PTX used in this study, the BRAF inhibitor vemurafenib^39^, the CDK4/6 inhibitor palbociclib^40^, and temozolomide^41^ can all induce cell senescence in melanoma cells. The reasons why melanoma cells preferentially adopt senescence are largely unknown. As a tumour microenvironment is in an immunosuppressive state, therapy-induced senescent cells may not be eliminated timely, thus resulting in the accumulation of SASP factors. Therefore, targeting the senescent cells, the key SASP factors or the reproductive signalling pathway using senolytic drugs^42^, neutralising antibodies or pathway inhibitors may be a reasonable strategy to improve the therapeutic effect in melanoma chemotherapy.

## Materials and methods

### Cell culture and reagents

The cell lines, including A375, A549, HeLa, B16F10 and MDA-MB-231, were purchased from the Type Culture Collection of the Chinese Academy of Sciences, Shanghai, China. Other melanoma cell lines, including A875, M14, OCM-1A and SK-MEL-2 were obtained from iCell Bioscience Inc., Shanghai, China. All the cells were cultured in DMEM or RPMI 1640 medium (Gibco, Carlsbad, CA, USA) containing 10% FBS (Hyclone, USA). CDDP (P4394) and dacarbazine (D2390) came from Sigma-Aldrich Co., USA. PTX (SP8020) was purchased from Solarbio Science and Technology Co., Ltd, China. Dacarbazine stock solution was prepared using 1 M HCl and exposed to light for 1 h before use. The PH was adjusted with NaOH after dacarbazine was diluted with a growth medium^43^.

The following antibodies were purchased from Cell Signalling Technology (Beverly, MA, USA): p-Akt (Ser473) (#9271), Akt (pan) (#2920), p-(Erk1/2) (Thr202/Tyr204) (#9101), Erk1/2 (#9102), p-P65 (Ser536) (#3033), P65 (NF-κB) (#8242), p-P53(Ser15) (#9284), P53 (#9282S), p-JNK (Thr183/Tyr185) (#4668), JNK (#9252), p-MK2 (Thr334) (#3041), MK2 (#3042), PARP1 (#9532), p-Histone H2A.X (#2577), p21 Waf1/Cip1 (#2946), p-Rb (Ser807/811) (#9308), IκBα (#4812) and FRA1 (#5281). The antibodies of p16INK4a (ab81278) and DDB2 (ab181136) were obtained from Abcam PLC. (Cambridge, UK). Anti-IL-8 (MAB208), anti-IL-1α (MAB200) and the cytokines IL-1α (200-LA) and IL-8 (208-IL) were purchased from R&D System (Minneapolis, MN, USA). α-tubulin (T5168) was obtained from Sigma-Aldrich (St. Louis, MO, USA).

### Quantitative reverse transcription PCR

Total RNA was extracted using TRIZOL reagent (Life Technologies, USA) and inversely transcribed into cDNA using the PrimeScript RT reagent kit with gDNA Eraser (RR047A, TaKaRa, Japan). Quantitative PCR reaction was conducted using SYBR Select Master Mix (4472908, Applied Biosystems, USA) in LightCycler 96 System (Roche, USA). GAPDH served as the internal reference gene.

### Western blot and immunofluorescence detection

Cell lysates were collected and the protein concentration was determined. Protein samples were separated using sodium dodecyl sulphate polyacrylamide gel. The PVDF (polyvinylidene fluoride) membrane was blocked using 5% milk and then incubated with primary antibodies overnight at 4 ℃. After incubating with HRP (horseradish peroxidase)-conjugated secondary antibodies, the membrane was exposed using the Azure c400 imaging system (Azure Biosystems, Inc., USA). For immunofluorescence, the cells were fixed using 4% paraformaldehyde and then permeabilised and blocked using 4% BSA (bovine serum albumin) containing goat serum and Triton X-100. After incubation with primary and fluorescent secondary antibodies, the cells were observed under a fluorescence microscope.

### Sample preparation and ELISA

To prepare the senescent conditioned medium (Sen CM), A375 cells were treated with CDDP (2 and 4 μM) for 24 h. At 4 or 7 days after the CDDP treatment, one dish was used for cell counting, and the others were cultured with DMEM medium containing 0.5% FBS. 24 h later, the supernatant was filtered and cryopreserved in −80 °C for further use. For the non-senescent conditioned medium (NS CM), normal A375 cells were synchronously treated with a vehicle. After 4 or 7 days, the same cell amount as that of senescent cells was seeded in 0.5% FBS medium and CM was collected 24 h later. Pre CM was prepared by treating A375 cells with CDDP (4 μM) for 24 h. Then, the cells were cultured in a fresh medium for 3 h before being passaged, counted and cultured using 0.5% FBS medium. After another 24 h, the Pre CM was collected. To prepare tissue lysates, mice tumours were frozen with liquid nitrogen and then cut into pieces. Phosphate-buffered saline (PBS) containing protease inhibitors was added into tissue at a ratio of 7 μl/mg tissue. The tissues were then lysated mechanically. The ELISA procedure followed the instruction of the IL-8 (EK0413) and IL-1α (EK0389) ELISA Kit provided by Boster Biological Technology, Co. Ltd., China.

### Clone formation assay (CFA) and alamarBlue assay

For CFA, 500 cells per well were seeded into six-well plate in duplicate for each group. 24 h later, NS CM, Sen CM or DMEM with 0.5% FBS was premixed with an equal volume of DMEM containing different concentrations of serum (0.5, 1.5 or 20%) and then added into the plates. When the clones became evident, the cells were stained with 1% crystal violet.

AlamarBlue assay was performed according to the manufacturer’s guidelines (DAL1025, Thermo Fisher Scientific, USA). Briefly, about 2000 cells per well were seeded into a 96-well plate with five repetitions in each group. 24 h later, the CMs or DMEM medium was added into the plates. At different intervals, alamarBlue was added and fluorescence intensity was detected using a microplate reader.

### Gene array

A375 cells were seeded in triplicate and serum starved for 48 h. Then, DMEM medium with 0.25% FBS (negative control, NC), DMEM+ NS CM (1:1) and DMEM+ Sen CM (1:1) was added into one of the three wells, respectively, and cultured for 9 h. The cells were collected and mRNA array was performed using Affymetrix PrimeView Human Gene Expression Array (USA). Differential genes with a fold change more than 2 or 1.5 times were collected for further analysis.

### In vivo experiment in mice tumour xenograft

The BALB/c nude mice and C57BL/6 mice were obtained from the Center of Experimental Animals, Sun Yat-sen University, China. All the experimental procedures followed the institutional guidelines for the care and use of laboratory animals in Guangdong Medical University, China. To produce tumour xenograft, human A375 and mice B16F10 cells were suspended with PBS and subcutaneously injected into the right back of the nude mice and C57BL/6 mice, respectively. To investigate cell senescence and SASP in vivo, CDDP was intraperitoneally injected into nude mice once at a dose of 60 μg/10 g weight. For B16F10 tumour xenograft in C57BL/6 mice, dacarbazine (800 μg/10 g) and CDDP (200 μg/10 g) was successively injected with an interval of 3 h. In the antibody neutralisation experiment, after the tumour formation and CDDP delivery, about 4 μg antibody (anti-IL-1α or anti-IL-8) or the same volume of the vehicle was intratumour injected twice. After the mice were euthanatised, the tumour tissue was weighed and freezing sliced or lysated for further detection. Mice serum was collected by enucleation of the eyeball and used for ELISA detection.

### Statistical analysis

SPSS 19.0 software was used to analyse the results. Data are expressed as mean ± SEM (number of observations). Student’s t test or ANOVA and then LSD test were used to evaluate the difference. *P* < 0.05 was considered significant.

## Electronic supplementary material

Supplementary Table 1

Supplementary Figures 1-10
