# Supplementary material for: Senescence-associated secretory factors induced by cisplatin in melanoma cells promote non-senescent melanoma cell growth through activation of the ERK1/2-RSK1 pathway
Source: Cell Death Dis. 2018 Feb 15;9(3):260. doi: 10.1038/s41419-018-0303-9 (PMC5833767; doi:10.1038/s41419-018-0303-9)
Supplement: Supplementary file 1 — Supplementary Table 1 [file 41419_2018_303_MOESM1_ESM.docx]

**Supplementary Table 1.** The differentially expressed genes in A375 cells induced by Sen CM *vs.* NS CM with fold change more than 1.5 times.

| Up-regulated Genes | Entrez Gene ID | Ref | Down-regulated Genes | Entrez  Gene ID | Ref |
| --- | --- | --- | --- | --- | --- |
| C15orf48 | 84419 |  | CITED1 | 4435 |  |
| CCL2 | 6347 | [^1^](#_ENREF_1)^,^ [^2^](#_ENREF_2) | CITED2 | 10370 | [^3^](#_ENREF_3) |
| CCL20 | 6364 | [^4^](#_ENREF_4)^,^ [^5^](#_ENREF_5) | GPR110 | 266977 |  |
| CXCL1 | 2919 | [^5^](#_ENREF_5) | HIST1H1D | 3007 |  |
| CXCL2 | 2920 | [^6^](#_ENREF_6) | HMGCS1 | 3157 |  |
| CXCL3 | 2921 | [^2^](#_ENREF_2) | INSIG1 | 3638 | [^7^](#_ENREF_7) |
| CXCL8 | 3576 | [^5^](#_ENREF_5) | LOC100130872 | 10417 |  |
| ESM1 | 11082 | [^8^](#_ENREF_8) Δ | MEF2C | 4208 | [^9^](#_ENREF_9) |
| FDCSP | 260436 |  | RGS16 | 6004 | [^10^](#_ENREF_10) ∇ |
| ID1 | 3397 | [^11^](#_ENREF_11) | RGS3 | 5998 | [^12^](#_ENREF_12) ∇ |
| ID3 | 3399 |  | SOCS2 | 8835 |  |
| IRF9 | 10379 |  | SPON2 | 100130872 |  |
| KIAA0040 | 9674 |  |  |  |  |
| KRT6B | 3854 | [^13^](#_ENREF_13) |  |  |  |
| LCN2 | 3934 | [^14^](#_ENREF_14) Δ |  |  |  |
| PCDH9 | 5101 |  |  |  |  |
| PTX3 | 5806 | [^15^](#_ENREF_15) |  |  |  |
| SHISA2 | 387914 |  |  |  |  |
| SLC30A1 | 7779 | [^16^](#_ENREF_16) Δ |  |  |  |

The references (Ref) demonstrated that the genes could be regulated by ERK1/2 pathway, or on the contrary, regulating ERK1/2 pathway. Δ indicates the genes regulating ERK1/2 pathway positively. ∇ indicates the genes regulating ERK1/2 pathway negatively.

**Supplementary References:**

1. Hu WT, Li MQ, Liu W, Jin LP, Li DJ, Zhu XY. IL-33 enhances proliferation and invasiveness of decidual stromal cells by up-regulation of CCL2/CCR2 via NF-kappaB and ERK1/2 signaling. *Molecular human reproduction* 2014, **20**(4)**:** 358-372.

2. Kruger K, Schrader K, Klempt M. Cellular Response to Titanium Dioxide Nanoparticles in Intestinal Epithelial Caco-2 Cells is Dependent on Endocytosis-Associated Structures and Mediated by EGFR. *Nanomaterials (Basel, Switzerland)* 2017, **7**(4).

3. He Z, Leong DJ, Zhuo Z, Majeska RJ, Cardoso L, Spray DC*, et al.* Strain-induced mechanotransduction through primary cilia, extracellular ATP, purinergic calcium signaling, and ERK1/2 transactivates CITED2 and downregulates MMP-1 and MMP-13 gene expression in chondrocytes. *Osteoarthritis and cartilage* 2016, **24**(5)**:** 892-901.

4. Sandri S, Hatanaka E, Franco AG, Pedrosa AM, Monteiro HP, Campa A. Serum amyloid A induces CCL20 secretion in mononuclear cells through MAPK (p38 and ERK1/2) signaling pathways. *Immunology letters* 2008, **121**(1)**:** 22-26.

5. Wu HH, Hwang-Verslues WW, Lee WH, Huang CK, Wei PC, Chen CL*, et al.* Targeting IL-17B-IL-17RB signaling with an anti-IL-17RB antibody blocks pancreatic cancer metastasis by silencing multiple chemokines. *The Journal of experimental medicine* 2015, **212**(3)**:** 333-349.

6. Xander P, Brito RR, Perez EC, Pozzibon JM, de Souza CF, Pellegrino R*, et al.* Crosstalk between B16 melanoma cells and B-1 lymphocytes induces global changes in tumor cell gene expression. *Immunobiology* 2013, **218**(10)**:** 1293-1303.

7. Keeton AB, Bortoff KD, Franklin JL, Messina JL. Blockade of rapid versus prolonged extracellularly regulated kinase 1/2 activation has differential effects on insulin-induced gene expression. *Endocrinology* 2005, **146**(6)**:** 2716-2725.

8. Rocha SF, Schiller M, Jing D, Li H, Butz S, Vestweber D*, et al.* Esm1 modulates endothelial tip cell behavior and vascular permeability by enhancing VEGF bioavailability. *Circulation research* 2014, **115**(6)**:** 581-590.

9. Gao W, Pan B, Liu L, Huang X, Liu Z, Tian J. Alcohol exposure increases the expression of cardiac transcription factors through ERK1/2-mediated histone3 hyperacetylation in H9c2 cells. *Biochemical and biophysical research communications* 2015, **466**(4)**:** 670-675.

10. Choi CY, Rho SB, Kim HS, Han J, Bae J, Lee SJ*, et al.* The ORF3 protein of porcine circovirus type 2 promotes secretion of IL-6 and IL-8 in porcine epithelial cells by facilitating proteasomal degradation of regulator of G protein signalling 16 through physical interaction. *The Journal of general virology* 2015, **96**(Pt 5)**:** 1098-1108.

11. Cook PJ, Thomas R, Kingsley PJ, Shimizu F, Montrose DC, Marnett LJ*, et al.* Cox-2-derived PGE2 induces Id1-dependent radiation resistance and self-renewal in experimental glioblastoma. *Neuro-oncology* 2016, **18**(10)**:** 1379-1389.

12. Liu Y, Huang H, Zhang Y, Zhu XY, Zhang R, Guan LH*, et al.* Regulator of G protein signaling 3 protects against cardiac hypertrophy in mice. *Journal of cellular biochemistry* 2014, **115**(5)**:** 977-986.

13. Sizemore GM, Sizemore ST, Seachrist DD, Keri RA. GABA(A) receptor pi (GABRP) stimulates basal-like breast cancer cell migration through activation of extracellular-regulated kinase 1/2 (ERK1/2). *The Journal of biological chemistry* 2014, **289**(35)**:** 24102-24113.

14. Ye D, Yang K, Zang S, Lin Z, Chau HT, Wang Y*, et al.* Lipocalin-2 mediates non-alcoholic steatohepatitis by promoting neutrophil-macrophage crosstalk via the induction of CXCR2. *Journal of hepatology* 2016, **65**(5)**:** 988-997.

15. Zhang J, Koussih L, Shan L, Halayko AJ, Chen BK, Gounni AS. TNF up-regulates Pentraxin3 expression in human airway smooth muscle cells via JNK and ERK1/2 MAPK pathways. *Allergy, asthma, and clinical immunology : official journal of the Canadian Society of Allergy and Clinical Immunology* 2015, **11:** 37.

16. Jirakulaporn T, Muslin AJ. Cation diffusion facilitator proteins modulate Raf-1 activity. *The Journal of biological chemistry* 2004, **279**(26)**:** 27807-27815.
