# Supplementary material for: Senescence-associated secretory factors induced by cisplatin in melanoma cells promote non-senescent melanoma cell growth through activation of the ERK1/2-RSK1 pathway
Source: Cell Death Dis. 2018 Feb 15;9(3):260. doi: 10.1038/s41419-018-0303-9 (PMC5833767; doi:10.1038/s41419-018-0303-9)
Supplement: Supplementary file 2 — Supplementary Figures 1-10 [file 41419_2018_303_MOESM2_ESM.docx]

**Supplementary Figures**

**Supplementary Figure 1**


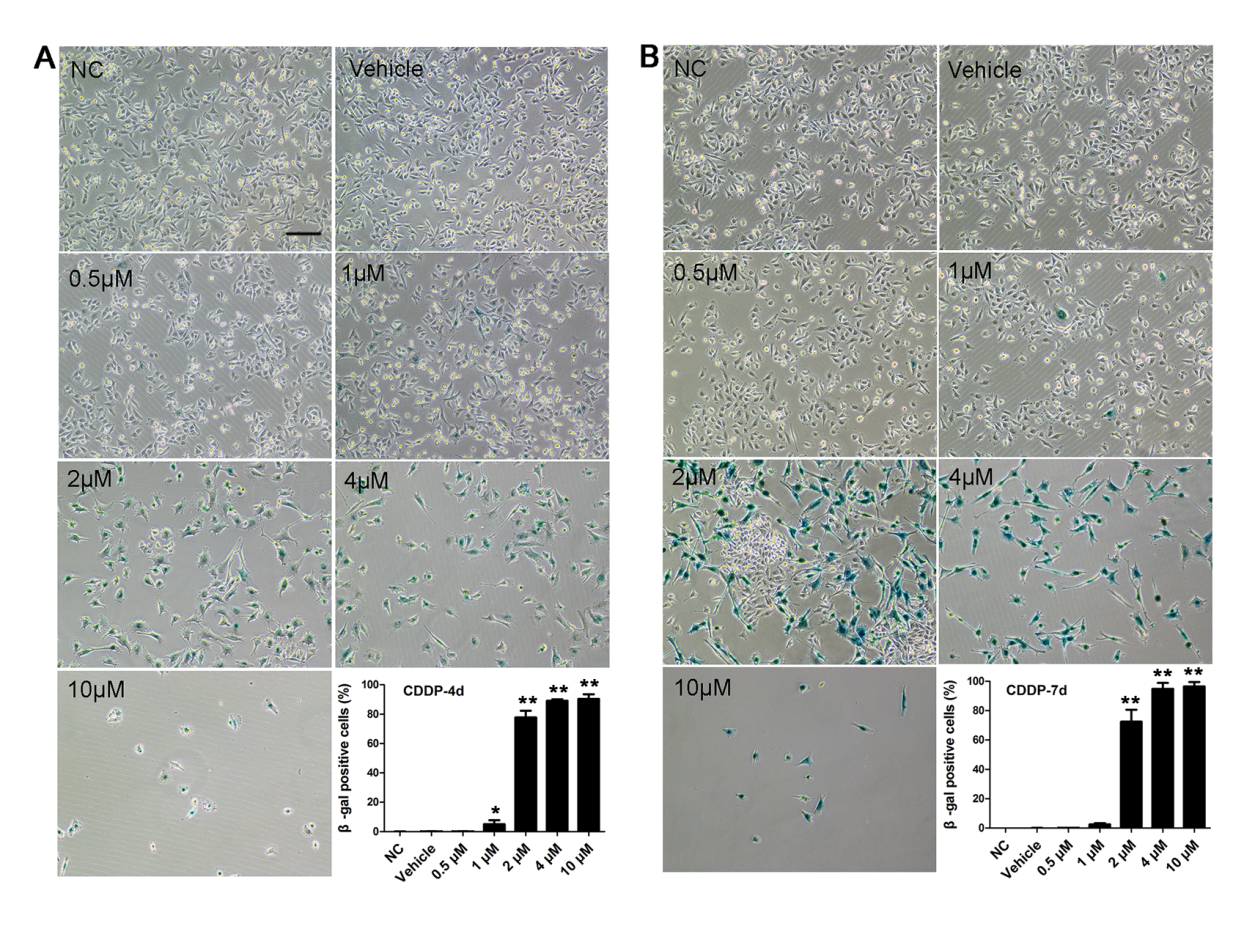


## Supplementary Figure 1. CDDP induced A375 cell senescence in a concentration-dependent manner. CDDP of various concentrations (0.5-10 μM) was added into growth medium (0 day) to incubate for 24 hours, and then completely replaced by fresh growth medium. After four days (A) or seven days (B) of CDDP treatment, β-gal staining was performed and the blue-stained cells were calculated. Bar is 100 μm. Data were derived from three independent experiments. **P*<0.05, ***P*<0.01 *vs.* NC (*n*=3).

**Supplementary Figure 2**


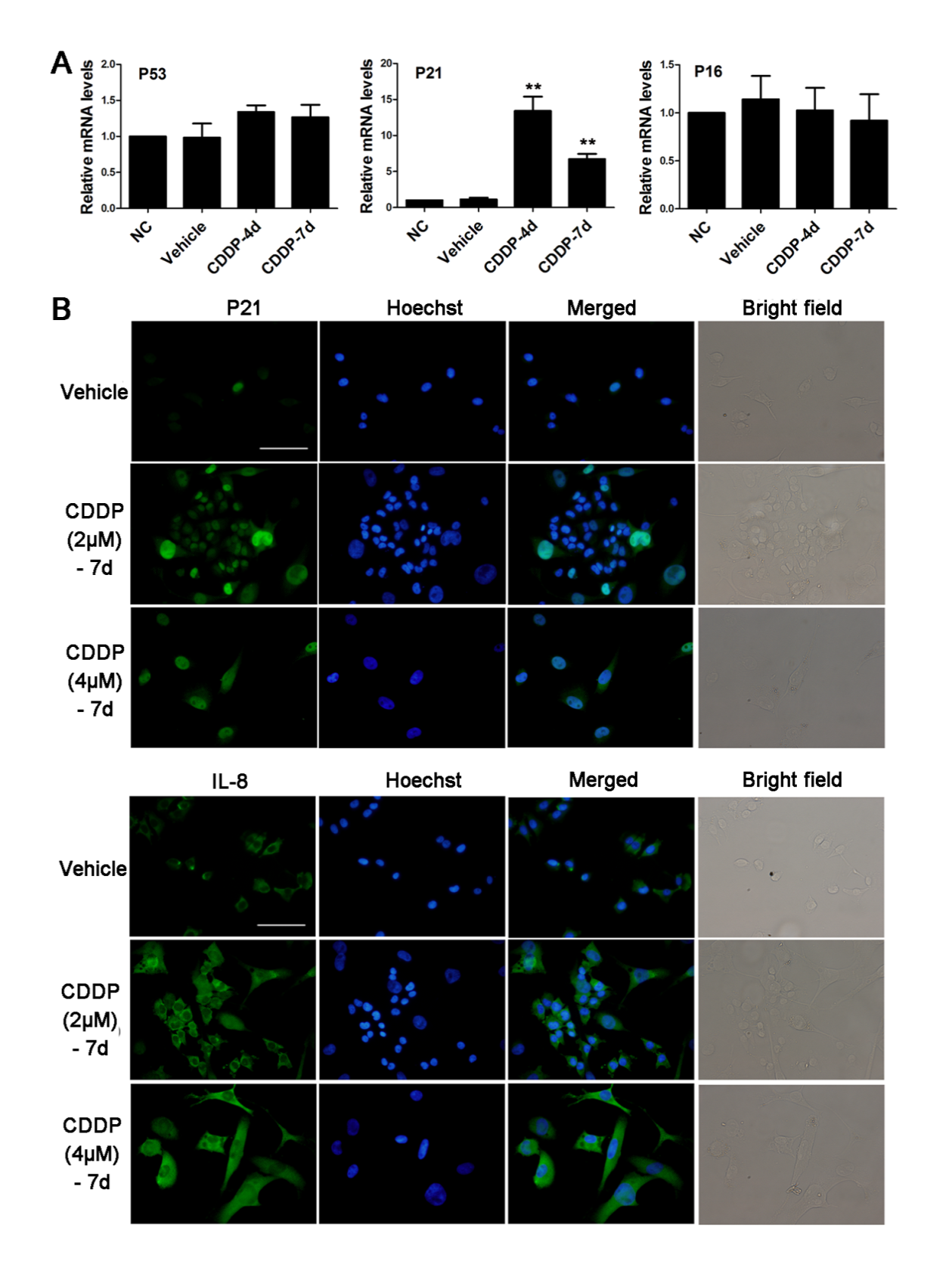
**Supplementary Figure 2.** CDDP enhanced the expression of P21 and IL-8 in A375 cells detected by qRT-PCR or immunofluorescence. **A,** CDDP (2 μM) increased the mRNA level of P21, but not that of P16 and P53 at four or seven days after treatment. **B,** CDDP (2 or 4 μM) increased the fluorescence intensity of P21 and IL-8 at seven days after treatment, which was located in nucleuses and cytoplasm, respectively. The bar is 100 μm. Data were from three independent experiments. ***P*<0.01 *vs.* NC (*n*=3).

**Supplementary Figure 3**


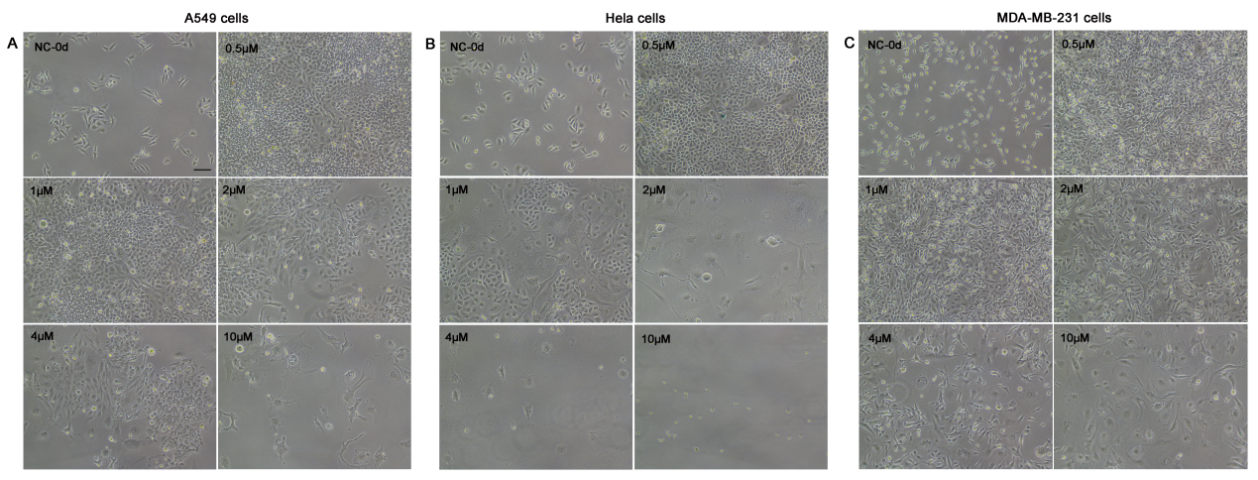


## Supplementary Figure 3. CDDP treatment in various concentrations could not obviously enhance β-gal staining in lung cancer A549, cervical carcinoma HeLa and breast carcinoma MDA-MB-231 cells. CDDP (0.5-10 μM) was added into growth medium of A549 cells (A), HeLa cells (B) and MDA-MB-231 cells (C) as described in Supplementary Figure 1. After seven days of the treatment, β-gal staining was performed. Negative control (NC) is the intact A375 cells before CDDP treatment (0 day). Bar is 100 μm.

**Supplementary Figure 4**


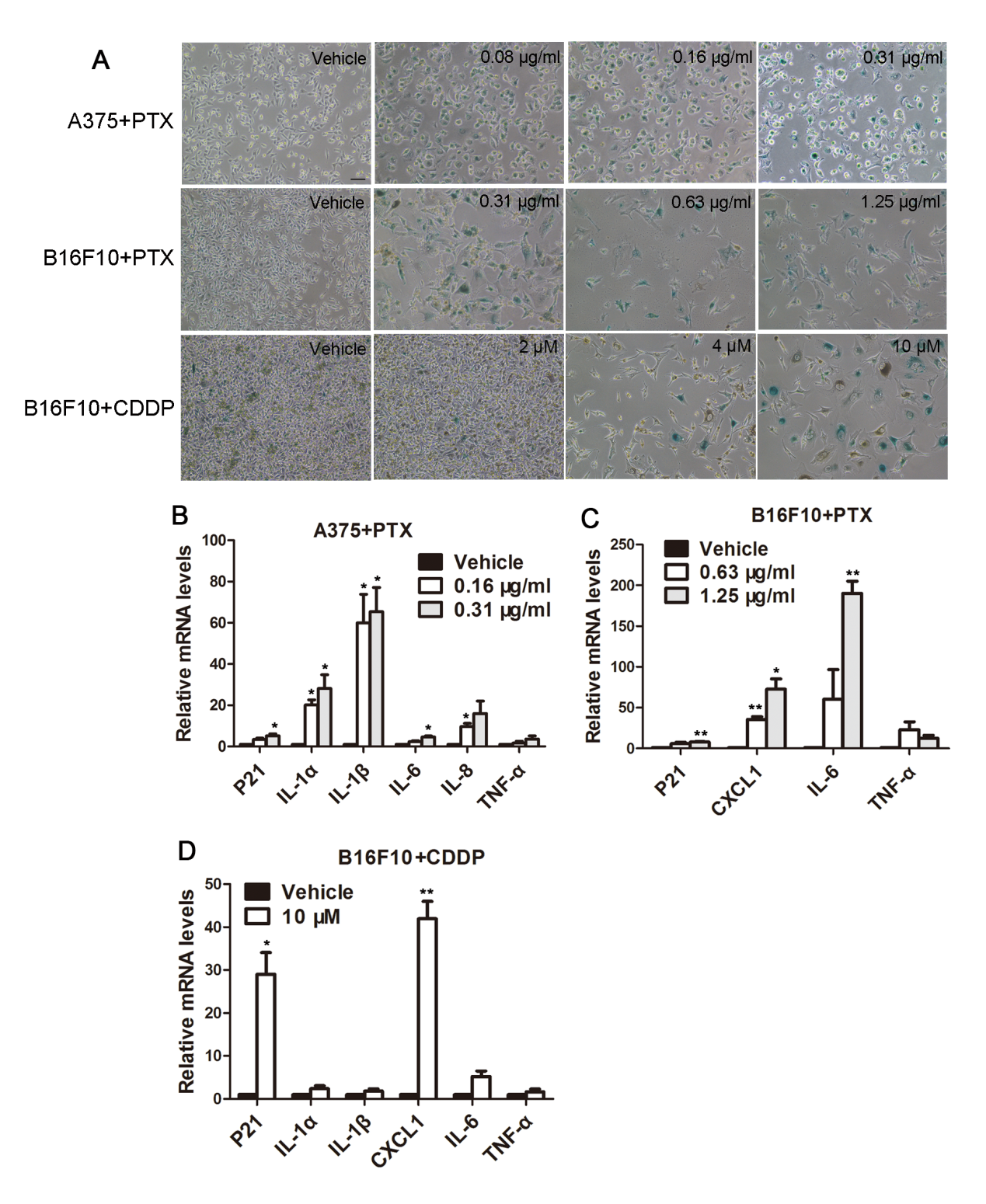
**Supplementary Figure 4.** Paclitaxel (PTX) and CDDP induced obvious senescence and SASP in melanoma B16F10 or A375 cells. Paclitaxel or CDDP was added into grow medium as described in Supplementary Fig. S1. After seven days, β-gal activity and the mRNA expression of P21 and SASP genes were detected. **A,** A375 and B16F10 cells showed differential sensitivity to chemotherapeutics. 0.08-0.31 μg/ml and 0.31-1.25 μg/ml of PTX were used for A375 and B16F10 cells, respectively, to induce cell senescence. CDDP at 2-10 μM was used in B16F10 to induce cell senescence. Blue-stained cells emerged with a concentration-dependent manner in these experiments. Furthermore, PTX induced consistent mRNA upregulation of P21 and the SASP genes in both A375 cells (**B**) and B16F10 cells (**C**). Similarly, CDDP (10 μM) induced obvious upregulation of P21 and SASP genes in B16F10 cells (**D**). Bar is 100 μm. **P*<0.05, ***P*<0.01 *vs.* Vehicle (*n*=3).

**Supplementary Figure 5**


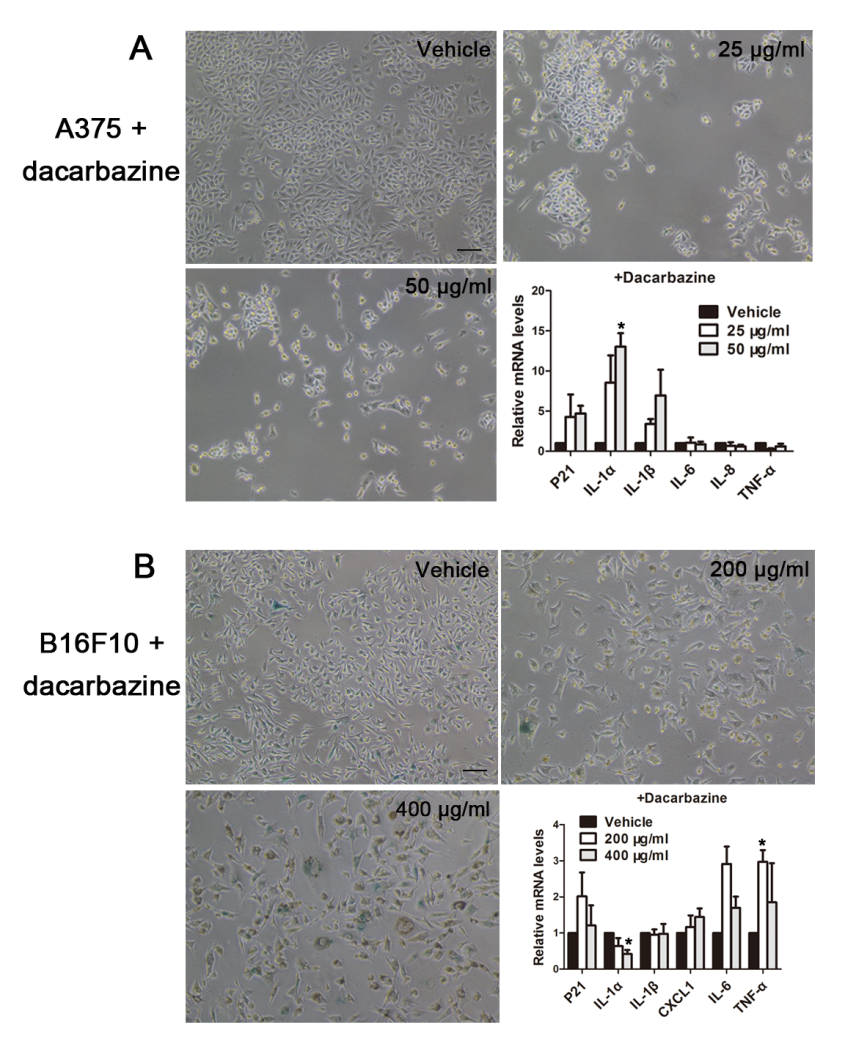


## Supplementary Figure 5. Dacarbazine induced aberrant upregulation of SASP genes but did not evidently enhance β-gal staining in melanoma B16F10 and A375 cells. A, Dacarbazine (25-50 μg/ml) exerted only a marginal effect on β-gal activity in A375 cells though substantially upregulated the mRNA levels of P21, IL-1α and IL-1β (*n*=3). B, Dacarbazine (200-400 μg/ml) exerted very weak effect on β-gal activity while obviously increased the mRNA levels of P21, IL-6 and TNF-α in B16F10 cells. Bar is 100 μm. **P*<0.05 *vs.* Vehicle (*n*=3).

**Supplementary Figure 6**


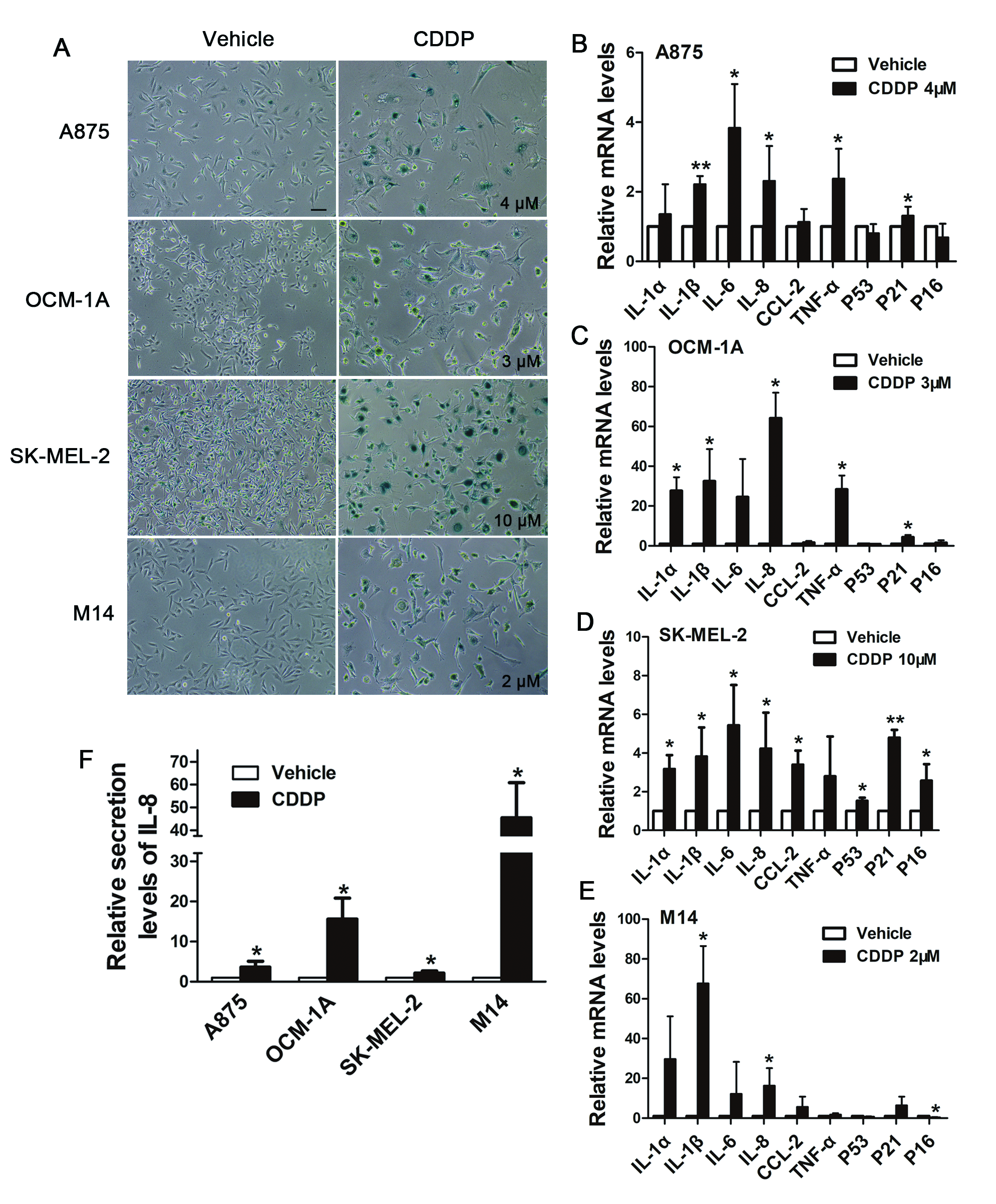


**Supplementary Figure 6.** CDDP induced senescence and SASP in several human melanoma cell lines. The method of senescence induction was similar to that of Figure 1A. **A,** CDDP induced cell enlargement and β-gal staining in the four cell lines seven days after the treatment. The concentrations of CDDP have been optimized and the optimal concentrations are indicated in the figures. **B-E,** CDDP generally promoted the mRNA expression of the senescence-related and SASP genes in A875, OCM-1A, SK-MEL-2 and M14 cell lines seven days after the treatment. **F,** CDDP increased the secretion of IL-8, evaluated by ELISA using the CMs collected on the eighth day. The bar is 100 μm. **P*<0.05, ***P*<0.01 *vs.* Vehicle (*n*=3).

**Supplementary Figure 7**


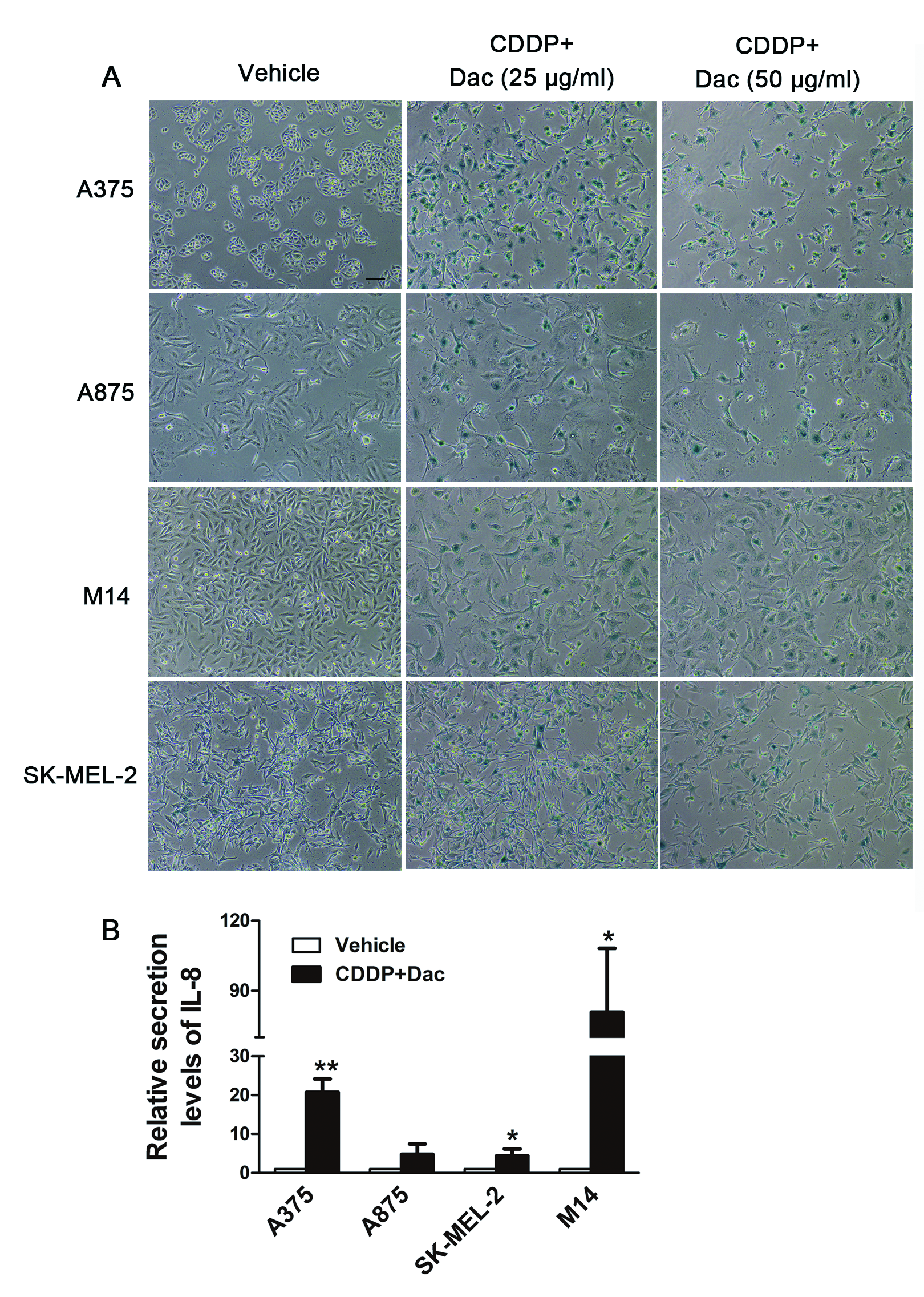


**Supplementary Figure 7.** CDDP and dacarbazine together induced cell senescence and SASP in melanoma cell lines. CDDP (2 μM) and dacarbazine (Dac) were simultaneously added into the growth medium of A375, A875, SK-MEL-2 or M14 melanoma cells, similar to the methods of Figure 1A. After seven or eight days, cells and CMs were collected and then β-gal staining (**A**) and ELISA detection of IL-8 (**B**) were performed. The bar is 100 μm. **P*<0.05, ***P*<0.01 *vs.* Vehicle (*n*=3).

**Supplementary Figure 8**


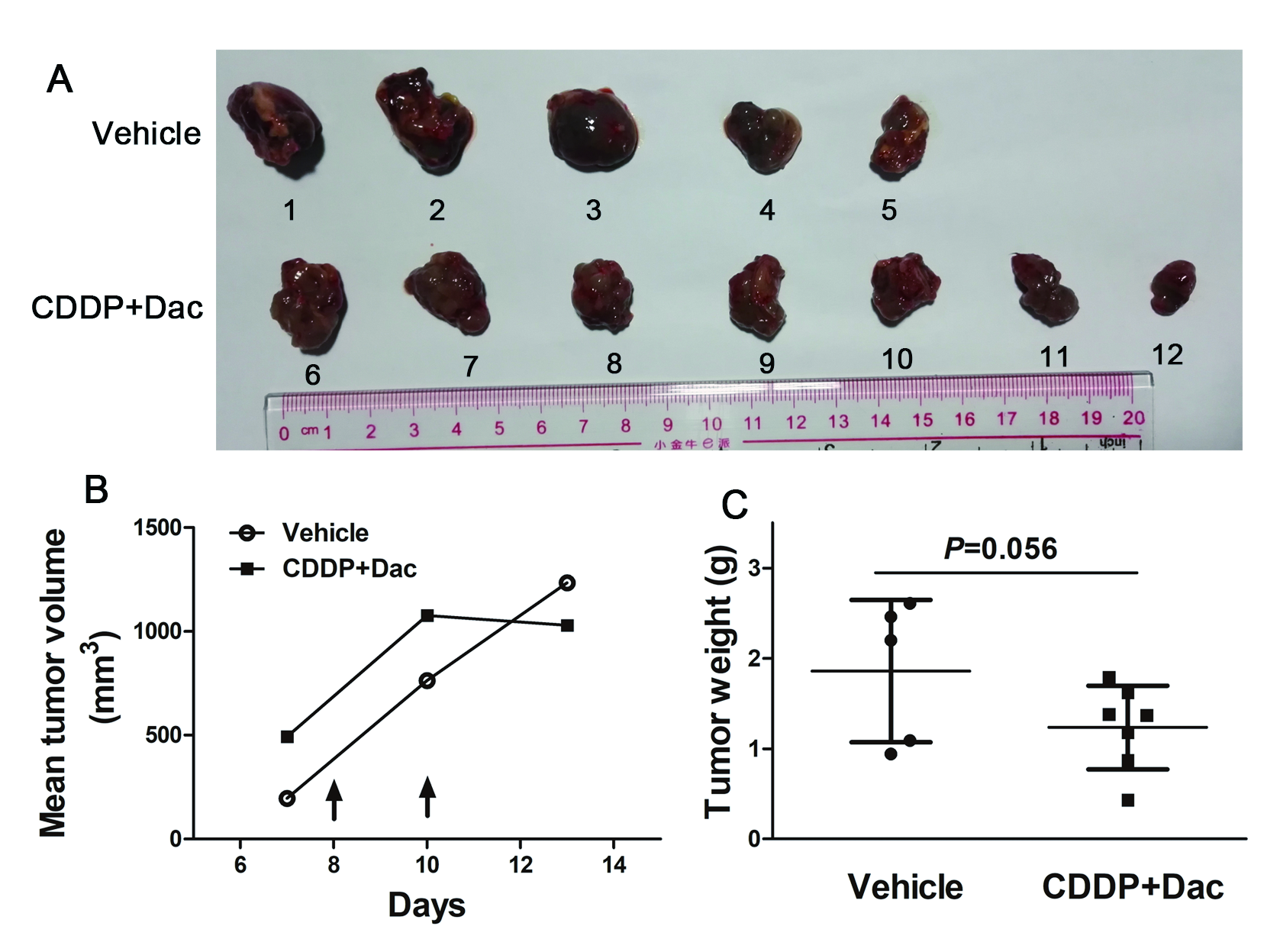


**Supplementary Figure 8.** The combined treatment of CDDP and dacarbazine inhibited tumour growth. After the subcutaneous injection of B16F10 cells (0 day) and the formation of tumour masses in C57BL/6 mice, both dacarbazine and CDDP were intraperitoneally injected twice on the eighth and tenth day (arrow, **B**). On the 13th day, tumour tissues were isolated and detected. **A,** Tumour tissues derived from the control (n=5) and treated (n=7) mice. **B,** Tumour growth curves based on mean tumour volume. **C,** Tumour weights of the two groups of mice.

**Supplementary Figure 9**


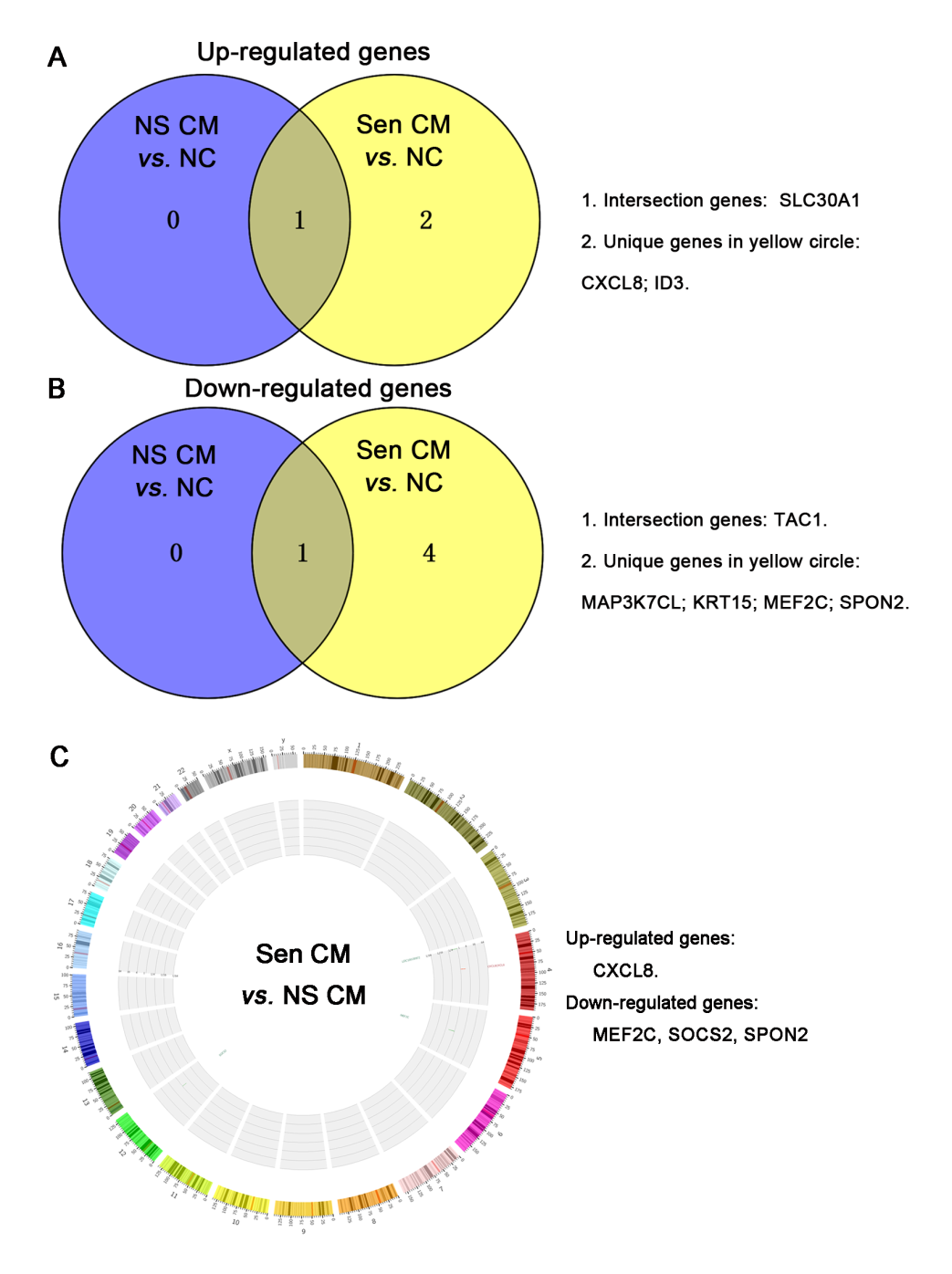


**Supplementary Figure 9.** The differentially expressed genes induced by Sen CM and NS CM in A375 cells screened using mRNA array. The genes with fold change more than 2 times were included in the analysis. Venn diagrams of the up-regulated (**A**) or down-regulated (**B**) genes induced by Sen CM and NS CM, in comparison with DMEM (NC). **C,** The differential genes regulated by Sen CM *vs.* NS CM in A375 cells.

**Supplementary Figure 10**


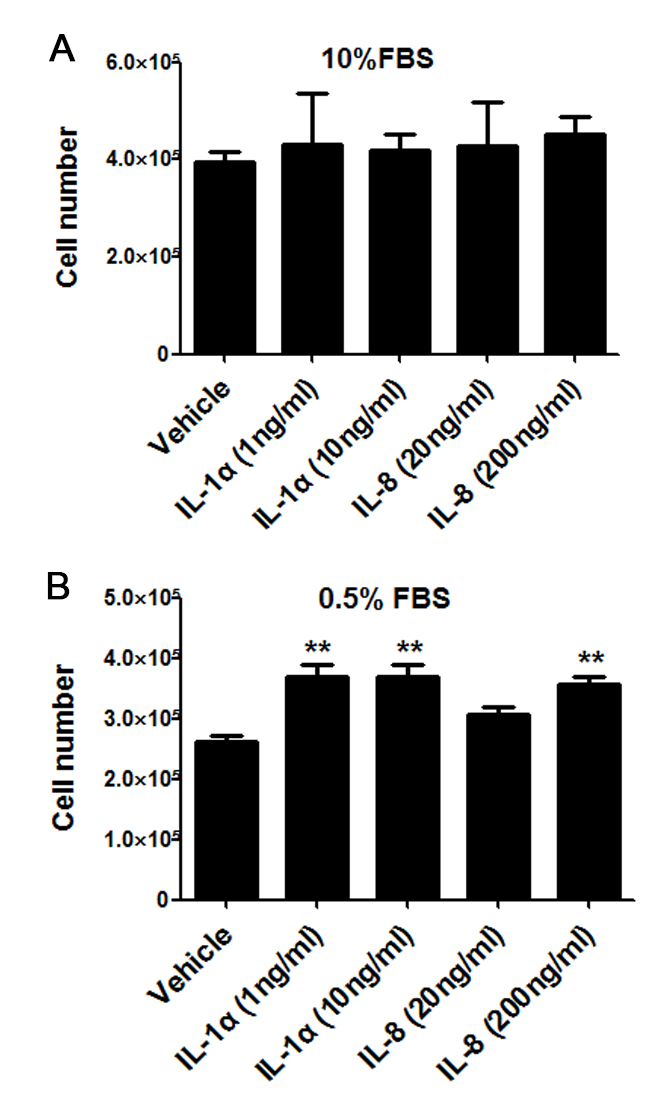


**Supplementary Figure 10.** IL-1α and IL-8 promoted A375 cells proliferation in 0.5% FBS medium. 4×10^3^ cells were seeded into 12-well plates in duplicate (0 day). 24 hours later, the growth medium was replaced with fresh medium containing 10% (**A**) or 0.5% (**B**) FBS, and then IL-1α or IL-8 was added. The growth medium together with IL-1α or IL-8 was refreshed every two days. At the fourth day, the cell number in each well was counted. Data were from four independent experiments. ** *P*<0.01 *vs.* Vehicle.
